# Expression conservation within the circadian clock of a monocot: natural variation at barley *Ppd-H1* affects circadian expression of flowering time genes, but not clock orthologs

**DOI:** 10.1186/1471-2229-12-97

**Published:** 2012-06-21

**Authors:** Chiara Campoli, Munqez Shtaya, Seth J Davis, Maria von Korff

**Affiliations:** 1 Max Planck Institute for Plant Breeding Research, Carl von Linné Weg 10, D50829, Cologne, Germany; 2 An-Najah National University, P. O. Box 7, Nablus, Palestinian Territories

**Keywords:** *Hordeum vulgare*, Circadian Clock, Photoperiod, Flowering, *Ppd-H1*

## Abstract

**Background:**

The circadian clock is an endogenous mechanism that coordinates biological processes with daily changes in the environment. In plants, circadian rhythms contribute to both agricultural productivity and evolutionary fitness. In barley, the photoperiod response regulator and flowering-time gene *Ppd-H1* is orthologous to the Arabidopsis core-clock gene *PRR7*. However, relatively little is known about the role of *Ppd-H1* and other components of the circadian clock in temperate crop species. In this study, we identified barley clock orthologs and tested the effects of natural genetic variation at *Ppd-H1* on diurnal and circadian expression of clock and output genes from the photoperiod-response pathway.

**Results:**

Barley clock orthologs *HvCCA1*, *HvGI*, *HvPRR1*, *HvPRR37* (*Ppd-H1*), *HvPRR73*, *HvPRR59* and *HvPRR95* showed a high level of sequence similarity and conservation of diurnal and circadian expression patterns, when compared to Arabidopsis. The natural mutation at *Ppd-H1* did not affect diurnal or circadian cycling of barley clock genes. However, the *Ppd-H1* mutant was found to be arrhythmic under free-running conditions for the photoperiod-response genes *HvCO1*, *HvCO2*, and the MADS-box transcription factor and vernalization responsive gene *Vrn-H1*.

**Conclusion:**

We suggest that the described eudicot clock is largely conserved in the monocot barley. However, genetic differentiation within gene families and differences in the function of *Ppd-H1* suggest evolutionary modification in the angiosperm clock. Our data indicates that natural variation at *Ppd-H1* does not affect the expression level of clock genes, but controls photoperiodic output genes. Circadian control of *Vrn-H1* in barley suggests that this vernalization responsive gene is also controlled by the photoperiod-response pathway. Structural and functional characterization of the barley circadian clock will set the basis for future studies of the adaptive significance of the circadian clock in *Triticeae* species.

## Background

The circadian clock is an autonomous oscillator that produces endogenous biological rhythms with a period of about 24 hours. This clock allows organisms to anticipate predicted daily changes in the environment and to coordinate developmental and metabolic processes with environmental cues, such as light and temperature, that cycle with the rotation of the earth [1-5]. Conceptually, a circadian system comprises three basic components: input pathways that sense light and temperature, a core oscillator that defines the rhythm, and output pathways that control various developmental and metabolic processes, resulting in the optimal adaptation to daily changing environments. The core oscillator that generates circadian rhythms is comprised of autoregulatory interlocking positive/negative feedback loops. In the eudicot Arabidopsis, the central loop called the “core oscillator” is composed of two partially redundant Myb-like transcription factors, CIRCADIAN CLOCK ASSOCIATED 1 (CCA1) [6] and LATE ELONGATED HYPOCOTYL (LHY) [7], and the pseudo response regulator (PRR) TIMING OF CAB EXPRESSION 1 (TOC1). The morning expressed CCA1 and LHY repress *TOC1* by directly binding to its promoter, which results in the evening accumulation of TOC1 that in turn represses *CCA1* and *LHY* expression [8]. These three genes are critical to sustain rhythms as the *cca1 lhy toc1* triple mutant was found to be arrhythmic [9]. The core oscillator is further fine-tuned by a morning-phased loop and an evening-phased loop. The morning loop includes members of the pseudo response regulated gene family, *PRR3, PRR5, PRR7* and *PRR9* which contain a pseudo receiver domain at the N terminus and a CCT (CONSTANS, CONSTANS-LIKE, and *TOC1*) motif at the C terminus [10,11]. *PRR* transcripts start accumulating after dawn sequentially in the order of *PRR9, PRR7, PRR5, PRR3,* and *PRR1/TOC1*[12], and it has been shown that *PRR7* and PRR9 repress *CCA1* and *LHY* during the day [10]. The evening-phased loop is proposed to include GIGANTEA (GI), ZEITLUPE (ZTL), *TOC1*, and an unknown factor Y. GI decreases *TOC1* protein level through stabilization of the ZTL protein [13,14]. The decreased *TOC1* protein tends to relieve repression of Y, increased Y expression in turn activates *TOC1* expression, so that Y directly and GI indirectly activate *TOC1* expression [15].

Output pathways from the oscillator convey circadian rhythms to the various physiological and molecular processes, which include many with agronomic importance, such as photosynthesis, growth, phytohormone signaling, and photoperiodic flowering [16,17]. The circadian clock may thus be a key for improving agronomic performance and stress adaptation of crops. Indeed, diurnal expression analysis of field grown maize showed that ~22% of all genes in leaf tissue exhibit diurnal expression patterns [18]. In addition, a null allele of the rice GI ortholog affected diurnal expression of 75% of all tested genes and conferred reduced seasonal adaptability in field grown rice [19]. These studies highlight the critical role of the clock in cereals. Some orthologs of Arabidopsis core-clock genes have been identified in the monocot plants rice [20] and *Lemna*[21,22]. The rice genome was reported to encode a single ortholog for *LHY* and *CCA1* and five *PRR* orthologs designated as *OsPRR1* orthologous to *TOC1*, Os*PRR73/OsPRR37* corresponding to *AtPRR7* or *AtPRR3,* and *OsPRR59/OsPRR95* corresponding to *AtPRR5* or *AtPRR9*[23]. Over-expression of *OsCCA1* or *OsPRR1* in Arabidopsis modified circadian rhythms [23]. Rice orthologs of *TOC1* and *PRR7* partially complemented the corresponding Arabidopsis *toc1* and *PRR7* mutants, which is consistent with the function of these proteins being conserved between monocots and Arabidopsis [20,24]. A full compendium analysis of the monocot clock awaits to be performed.

The temperate crop barley, which includes the domesticated form *Hordeum vulgare spp. vulgare* and the wild subspecies *Hordeum vulgare spp. spontaneum*, is characterized by high genetic diversity and good adaptation to stress prone marginal environments [25,26]. Interestingly, adaptation in barley is influenced by the photoperiod response gene *Ppd-H1*, also known as *HvPRR37*, which is orthologous to the rice gene *OsPRR37*[23] and the Arabidopsis clock gene *PRR7*[27]. A natural, recessive mutation in the CCT domain of *Ppd-H1* causes photoperiod insensitivity and late flowering in cultivated spring barley. In contrast, wild and cultivated winter barley genotypes harbor the photoperiod responsive *Ppd-H1* allele, which induces early flowering under long photoperiods. Barley genotypes with a photoperiod responsive *Ppd-H1* allele are characterized by elevated expression of *Vrn-H3 (HvFT1)* homologous to the Arabidopsis gene *FLOWERING LOCUS T (FT)*[27]. In Arabidopsis, FT is the mobile florigen hormone that moves as a protein from the leaves through the phloem to the shoot apical meristem where it induces the switch from vegetative to reproductive growth [28,29]. *FT* expression is triggered by the photoperiod response gene *CONSTANS (CO)*[30]. CO protein is degraded in darkness and expression of the protein during the day is crucial for induction of the floral activator *FT* and flowering [30]. It was suggested that the mutation in *Ppd-H1* of spring barley delayed flowering time by shifting the diurnal expression peaks of the barley *CO* orthologs *HvCO1* and *HvCO2* into the dark phase, so that the proteins are not synthesized and *Vrn-H3 (HvFT1)* not expressed [27].

Winter barley is vernalization sensitive, exposure to prolonged periods of cold during winter are translated into an increased competence to flower in spring. Vernalization response is controlled by variation at the vernalization genes *Vrn-H1* and Vrn-H2 and by the MADS-box transcription factors HvVrt2, HvBM1, and *HvBM10*, which are cereal orthologs of *SHORT VEGETATIVE PHASE (SVP)* in Arabidopsis [31,32]. In winter barley, *Vrn-H1*, with similarity to the Arabidopsis meristem identity genes *APETALA1, CAULIFLOWER*, and *FRUITFUL*, is only expressed after exposure to cold [33]. Insertions or deletions in the first intron of *Vrn-H1* in spring barley cause up-regulation of the gene independently of vernalization [34]. Spring barley is also characterized by a deletion of the entire *Vrn-H2* locus, which includes one truncated and two full sequence ZCCT (Zinc finger and CCT domain) genes with no clear orthologs in Arabidopsis [35]. In photoperiod-sensitive winter barley, *Vrn-H2* represses *Vrn-H3 (HvFT1)* to counteract the *Ppd-H1* dependent long day induction of *Vrn-H3* prior to winter. Up-regulation of *Vrn-H1* during vernalization and consequent down-regulation of *Vrn-H2* transcript levels in the leaf facilitate the up-regulation of *Vrn-H3* during long days mediated by *Ppd-H1*[36]. *HvVrt2, HvBM1,* and *HvBM10* are floral repressors, which may act downstream of *Vrn-H1* and *HvFT1* in barley, and thus, integrate light and temperature dependent regulation of flowering [31,37]. However, the effects of variation at *Ppd-H1* on circadian expression of photoperiod and vernalization response genes have not yet been analyzed. The natural mutation in the *Ppd-H1* gene may affect the photoperiod and vernalization pathways either by changing circadian timing of clock genes or by direct control of flowering time genes independently from its clock function.

 In this study, we analyzed whether orthologs of Arabidopsis clock genes are structurally and functionally conserved in the temperate crop and long-day plant barley. For this we 1) identified barley clock orthologs from available genomic databases and 2) analyzed their diurnal and circadian expression patterns in two barley genotypes differing at the photoperiod response gene and clock ortholog *Ppd-H1*. We showed that barley clock orthologs exhibit a high level of sequence similarity and conservation of expression profiles as compared to Arabidopsis and rice clock genes. The natural mutation at *Ppd-H1* did not affect expression of clock genes, but caused arrhythmicity of clock output genes *HvCO1, HvCO2*, and *Vrn-H1* under constant conditions. Our study provides a characterization of the compendium of barley clock genes under circadian conditions, and sets the basis to explore the effects of the circadian clock on performance in temperate crop species.

## Methods

### Plant material and growth conditions

The spring barley cultivar Scarlett and an introgression line S42IL-107 derived by crossing Scarlett with the wild barley accession ISR42-8 were used in this study [38,39]. Scarlett has a mutation in the CCT domain of *Ppd-H1* and is late flowering under LD [27]. The introgression line S42IL-107 harbors the photoperiod responsive *Ppd-H1* allele introgressed from wild barley and is early flowering under LD. Replicate plants of both genotypes were grown in soil in a controlled environment growth chamber (Conviron) at 20°C/18°C day/night with a photoperiod of 16-h light and 8-h darkness (LD treatment). After two weeks of LD treatment, replicate plants per genotype were harvested for a total of 24-h, starting at the transition to lights on in the morning (T0). Night samples were collected in the dark. After LD sampling, plants were released into continuous light at constant 20°C (LL treatment). Collection of leaf material was started at T16 and sampling was carried out for a total of 48-h. Under LD and LL, leaf material was sampled every 2-h, while at the end of the day and beginning of the night (or subjective nights) sampling was performed every hour. Each sample contains the second youngest leaves of three pooled plants. Samples were immediately frozen in liquid nitrogen and stored at −80°C until required.

### Isolation of clock orthologs

Sequences of *HvGI*, *Ppd-H1(PRR37)*, and *PRR7*3 were retrieved from the literature [27,40,41]. To identify orthologs of *CCA1/LHY, PRR1, PRR5,* and *PRR9* we used the barley database of DFCI Barley Gene Index Project (TIGR gene index project at http://compbio.dfci.harvard.edu/tgi/definitions.html). Expressed sequence tag (EST) sequences (and Tentative consensus (TC) sequences,) that had high sequence similarity with *OsCCA1* (Os08g0157600), *OsPRR1* (Os02g40510) and *TaPRR1* (AK333193), *OsPRR59* (Os11g05930) and *OsPRR95* (Os09g36220) were recovered. Primers were designed on the basis of the homologous EST sequences and used to amplify from a cDNA pool of Golden Promise a full-length cDNA for *HvCCA1* and a partial gene for *HvPRR1* (Additional file 1: Table S1). RNA extraction and cDNA synthesis were performed as described in the next paragraph. Amplifications of 2 μL f cDNA were conducted in a 20 μL PCR reaction volume containing 0.4 μM of each primer, 1 U of TAQ polymerase (GO Taq, Promega), 0.08 mM dNTP, 1.5 mM MgCl_2_. Amplification conditions were as follows: 98°C for 5 min, 35 cycles of 98°C (1 min), 56°C (30 sec) and 72°C (3 min), followed by an extension step at 72°C (7 min). Amplified fragments were cloned using the TOPO TA cloning kit (Invitrogen) following manufacturer instructions. For each gene, two independent clones were identified and sequenced. Sequence analysis, alignments, and in silico translations, were performed using programs within the Lasergene® 8 suite (Dnastar, Madison WI).

### Sequence analysis

Orthologous protein sequences for clock genes in Arabidopsis and monocots were retrieved from TAIR (http://www.arabidopsis.org/), NCBI (http://www.ncbi.nlm.nih.gov/) and Phytozome (http://www.phytozome.net/) databases. If no protein sequences were found, they were generated by virtually translating the mRNA sequences from the NCBI database (Lasergene® 8 suite, Dnastar, Madison WI). Accession numbers are listed in Table 1. For each gene family, sequences were aligned by the CLUSTALW method using the MEGA5 program [42]. The evolutionary distances were computed using the Poisson correction method [43]. All positions containing gaps and missing data were excluded. The phylogenetic analysis was performed using the neighbor-joining method within the MEGA5 package [44]. We used 10,000 trials to obtain bootstrap values. The alignments used for the phylogenetic analysis are available as Additional file 2: Figure S1, Additional file 3: Figure S2, Additional file 4: Figure S3.

**Table 1:** Genes used in this study (* = translation manually corrected; ** = contig not covering the full coding sequence, first part of the protein is missing)

Species	Gene name	Gene ID	Protein ID
*Arabidopsis thaliana*	*AtPRR1/TOC1*	AT5G61380	AT5G61380
	*AtPRR3*	AT5G60100	AT5G60100
	*AtPRR7*	AT5G02810	AT5G02810
	*AtPRR5*	AT5G24470	AT5G24470
	*AtPRR9*	AT2G46790	AT2G46790
	*AtGI*	AT1G22770	AT1G22770
	*AtCCA1*	AT2G46830	AT2G46830
	*AtLHY*	AT1G01060	AT1G01060
*Oryza sativa*	*OsPRR1/TOC1*	LOC_Os02g40510	LOC_Os02g40510
	*OsPRR37*	LOC_Os07g49460	LOC_Os07g49460
	*OsPRR73*	LOC_Os03g17570	LOC_Os03g17570
	*OsPRR59*	LOC_Os11g05930	LOC_Os11g05930
	*OsPRR95*	LOC_Os09g36220	LOC_Os09g36220
	*OsGI*	NM_001048755	Os01g0182600
	*OsCCA1*	Os08g0157600	NP_001061032
*Sorghum bicolor*	*SbPRR1/TOC1*	Sb04g026190	Sb04g026190
	*SbPRR37*	Sb06g014570	Sb06g014570
	*SbPRR73*	Sb01g038820	Sb01g038820
	*SbPRR59*	Sb05g003660	Sb05g003660
	*SbPRR95*	Sb02g030870	Sb02g030870
	*SbGI*	Sb03g003650	Sb03g003650
	*SbCCA1*	XM_002443845	XP_002443890*
*Zea mays*	*ZmPRR1/TOC1*	NM_001154351	NP_001147823
	*ZmPRR37*	LOC100280240	NP001146641
	*ZmPRR73*	EU952116	ACG24234
	ZmPRR59	GRMZM2G135446	GRMZM2G135446
	*ZmPRR95*	NM_001158064	NP_001151536
	*ZmGI*	BK006299	DAA06172
	*ZmCCA1*	LOC100192868	NP_001131529
*Triticum aestivum*	*TaPRR1/TOC1*	AK333193	*in silico*
	*TaPRR37*	DQ885766	ABL09477
	*TaPRR73*	Contig built from the following TC: G118.111D24F010720, TC37276, TC376302, TC377674, TC391788, TC392931, TC400719, TC418129, TC434756	*in silico*
	*TaPRR59*	Contig built from the following TC: BJ298369, G608.119D1, TC376876, TC393119, TC399029, TC411244, TaE05039B10R, WHE2989, wr1.pk0105.g4	in silico**
	*TaPRR95*	Contig built from the following TC: FGAS012265, TC373568, TC436795	*in silico*
	*TaGI*	AF543844	AAQ11738
	*TaCCA1*	HQ222606	ADW09013*
*Hordeum vulgare*	*HvPRR1/TOC1*	JN603243	
	*HvPRR37*	AY970701	AAY42109
	*HvPRR73*	AK376549	BAK07744
	*HvPRR59*	AK361360	BAJ92567
	*HvPRR95*	AK252005	*in silico*
	*HvGI*	AY740524	AAW66946
	*HvCCA1*	JN603242	
*Brachypodium*	*BdPRR1/TOC1*	Bradi3g48880	Bradi3g48880
	*BdPRR37*	Bradi1g16490	Bradi1g16490
	*BdPRR73*	Bradi1g65910	Bradi1g65910
	*BdPRR59*	Bradi4g24970	Bradi4g24970
	*BdPRR95*	Bradi4g36077	Bradi4g36077
	*BdGI*	Bradi2g05230	Bradi2g05230*
	*BdCCA1*	Bradi3g16510	Bradi3g16510*

### RNA extraction, cDNA synthesis and real time qRT-PCR

Total RNA was extracted from 100 mg of tissue using TRIZOL® reagent (Invitrogen) following manufacturer’s instructions, except for the addition of RNaseH, followed by a DNase treatment (final volume 100 μL). First strand cDNA synthesis was performed on 4 μL of total RNA using 100 U of SuperScriptTM II RT (Invitrogen) and 500 ng of poly-T primer and following manufacturer’s recommendations (final volume 40 μL). The resulting cDNA was diluted 1:4 in nuclease-free water and stored in aliquots at −20°C.

Real-Time-quantitative PCRs (qRT-PCR) were performed on cDNA samples using gene-specific primers (Additional file 1: Table S1). Amplifications were performed using 4 μL of cDNA, 0.5 U of GoTaq DNA polymerase (Promega), 0.2 mM dNTP, 2.5 mM MgCl_2_, 0.2 μM each primer, and 0.5 μL of EvaGreen (Biotium) in a final volume of 10 μL. Reactions were performed in a LightCycler480 (Roche) with the following amplification conditions: 95°C for 5 min, 45 cycles of 95°C (10 s), 60°C (10 s) and 72°C (10 s). Appropriate non-template controls were included in each 384-well PCR. Dissociation analysis was performed at the end of each run and the melting curves for each primer pair showed a single peak confirming the specificity of the reaction. The standard curves were prepared from a dilution series of plasmids containing the target fragments and subjected to qRT-PCR analysis with the respective cDNA samples. Starting amounts for each data point were calculated based on the titration curve for each target gene and the reference (*HvActin*) gene using the LightCycler 480 Software (Roche; version 1.5). Expression values shown in the figures represent the average ± standard deviation of 2 technical replicates of the ratio between the target and reference gene values.

## Results

### Phylogenetic analyses of the barley clock genes

Barley sequences with high similarity to *OsCCA1, OsPRR1, OsPRR59,* and *OsPRR95,* orthologous to *CCA1/LHY, PRR1, PRR5* and *PRR9* in Arabidopsis were recovered from the barley EST databases. Based on the tentative consensus sequences (TCs), we obtained a full-length clone for *HvCCA1* (accession number JN603242) and a partial sequence for *HvPRR1* (accession number JN603243) from Golden Promise cDNA. Barley gene sequences of *HvGI*[40], *HvPRR37* (*Ppd-H1*) [27], Hv*PRR7*3 [41] were retrieved from the gene banks. In order to examine the structural conservation of these potential circadian clock genes in barley, candidate clock sequences from barley were compared in multiple protein alignments to orthologous sequences from Arabidopsis and monocot species, including rice, Brachypodium, maize, sorghum, and wheat (Additional file 5: Table S2).

**Figure 1:**
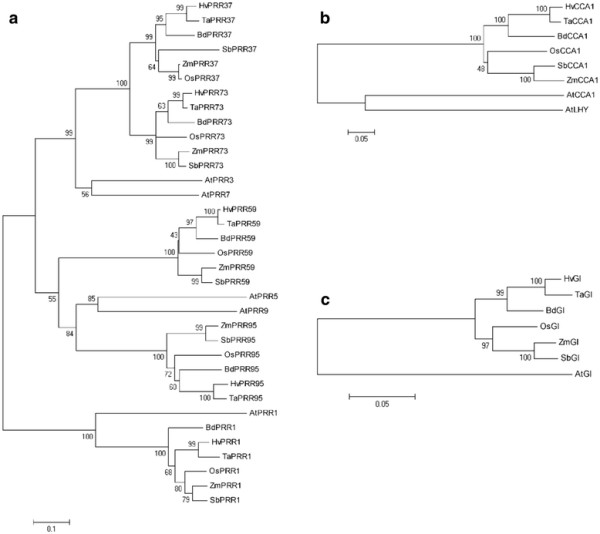
**Phylogenetic relationships of circadian clock related proteins.** Neighbor-joining phylogenetic trees were built from the protein alignments of the indicated sequences. **a**) PRR gene family, **b**) CCA1/LHY gene family, **c**) GI genes. Accession numbers are given in Table 1. Bootstrap values were calculated from 10,000 permutations.

In barley, five distinctive PRR sequences could be identified. These fell into three major clades, the PRR1/TOC1 clade, the PRR3/7 clade, and the PRR5/9 clade (Figure 1a). Orthologs of *OsPRR1* clustered with *PRR1/TOC1* from Arabidopsis and the PRR1-like genes could clearly be recognized as an outgroup of the PRR gene family. Each of the two remaining PRR clades showed two subgroups for monocot orthologs *OsPRR37, OsPRR7*3 and *OsPRR59, OsPRR95,* respectively. *AtPRR3* and *AtPRR7* were outgroups to the two monocot gene clusters PRR37 and *PRR7*3. *AtPRR5* and *AtPRR9,* however, grouped with the monocot PRR95 genes, while the monocot PRR59 sequences formed a separate cluster. Genetic distances were calculated from protein sequences for entire orthologous gene sequences or for domains within the monocot species and across the monocot species and Arabidopsis (Table 2). Variation in genetic distances between domains, genes and group of genes may indicate differences in evolutionary history and in the conservation of gene functions. Within the PRR genes, average genetic distances across Arabidopsis and monocots were lowest for the PRR1 gene group with a genetic distance of 0.39, followed by PRR3/7 with 0.51 and PRR5/9 with 0.86 (Table 2). The derived PRR amino-acid sequences were found to be most similar within their pseudo-receiver domain (mean genetic distance 0.32), and at the C-terminal end (mean genetic distance 0.22), which contains a CCT motif that is commonly also found in the CONSTANS (CO) family of flowering regulators [45] (Table 2, Additional file 4: Figure S3).

**Table 2:** Genetic distances for gene families across monocots and Arabidopsis for entire sequences or sequence domains

**Sequences**	**N° of sequences**	**Domains**	**Overall mean distances**
GI^a^	7	Nuclear localized protein	0.18
GI (monocots)^b^	6	Nuclear localized protein	0.10
CCA1/LHY	8	MYB transcription factor	0.55
CCA1 (monocot)	6	MYB transcription factor	0.27
AtCCA1 and AtLHY	7	MYB transcription factor	0.45
CCA1 and AtCCA1	7	MYB transcription factor	0.44
CCA1/LHY: MYB domain	8	MYB domain	0.05
CCA1/LHY: MYB domain (monocot)	6	MYB domain	0.01
PRRs	35	pseudoreceiver-CCT domain	0.87
PRR1	7	pseudoreceiver-CCT domain	0.39
PRR1 (monocots)	6	pseudoreceiver-CCT domain	0.22
PRR3/7	14	pseudoreceiver-CCT domain	0.51
PRR3/7 (monocots)	12	pseudoreceiver-CCT domain	0.46
PRR5/9	14	pseudoreceiver-CCT domain	0.86
PRR5/9 (monocots)	12	pseudoreceiver-CCT domain	0.90
PRRs: REC domain	35	Receiver domain	0.32
PRRs:REC domain (monocots)	30	Receiver domain	0.31
PRRs:CCT domain	35	CCT domain	0.22
PRRs:CCT domain (monocots)	30	CCT domain	0.22

^a^Arabidopsis and monocots.

^b^only monocots.

The identified *HvCCA1* protein sequence showed a high similarity to OsCCA1 and the redundant pair CCA1/LHY in Arabidopsis, as evidenced by the phylogenetic tree (Figure 1b). Sequence distances between *HvCCA1* and OsCCA1 were 0.26, while the overall mean distance across *CCA1* and LHY in Arabidopsis and the monocot species was 0.55 (Table 2, Additional file 5: Table S2). The *CCA1* protein sequences exhibited the highest sequence identity of 0.95 at the single MYB DNA-binding domain at its N-terminal end (Table 2, Additional file 3: Figure S2) [6,7].

Multiple sequence alignments using the GI proteins from the six monocot plants and Arabidopsis showed that the GI protein was characterized by the lowest overall mean sequence distance (0.18, Table 2, Figure 1c). *HvGI* was most similar to TaGI and BdGI with sequence distances of 0.03 and 0.07, respectively (Additional file 1c). *HvGI* was most similar to TaGI and BdGI with sequence distances of 0.03 and 0.07, respectively (Additional file 2: Figure S1, Additional file 5: Table S2). GI thus appears to be evolving slowly.

From our alignments we concluded that the barley sequences *HvCCA1* and *HvPRR1*, and the identified clones AK376549 (*HvPRR7*3), AK361360 (*HvPRR59*) and AK252005 (HvPRR95) were the likely orthologs of *CCA1/LHY, TOC1/PRR1, PRR3/7* and *PRR5/9* from Arabidopsis. In addition, mean distances calculated for protein sequences of the different clock genes across six different monocot species revealed that GI showed the highest conservation with a mean distance of 0.10, followed by PRR1 (0.22), and *CCA1* (0.27). The two clades of PRR37/73 and PRR59/95, which have evolved after the split of the eudicot and monocot lineages, showed the highest mean distances with 0.46 and 0.90, respectively.

### Diurnal and circadian expression patterns of putative barley clock orthologs

**Figure 2:**
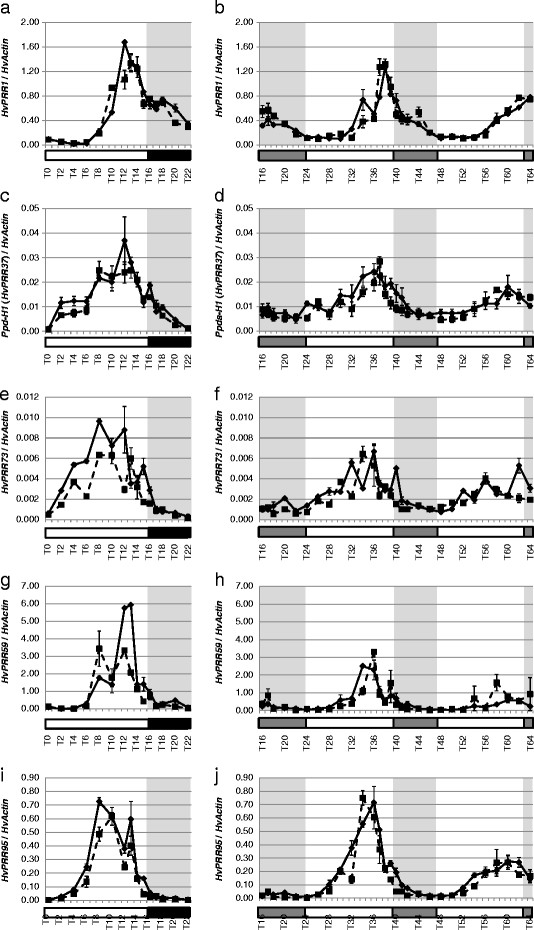
**Diurnal and circadian expression of PRR genes in the spring cultivar Scarlett (*ppd-H1 *) and the introgression line S42 IL-107 (*Ppd-H1 *) under long day and free running conditions.** Scarlett (solid line) and S42-IL107 (dashed line) were grown under long day conditions (16 hours light - left panel) for two weeks and then released in continuous light (right panel). Transcript accumulation was measured at two-hour intervals (one-hour interval at the end of the day and beginning of the night/subjective nights) by qRT-PCR analysis of specific genes and normalized to *HvActin*. Values represent the average of two technical replicates ± standard error. Black and grey bars indicate objective and subjective nights.

**Figure 3:**
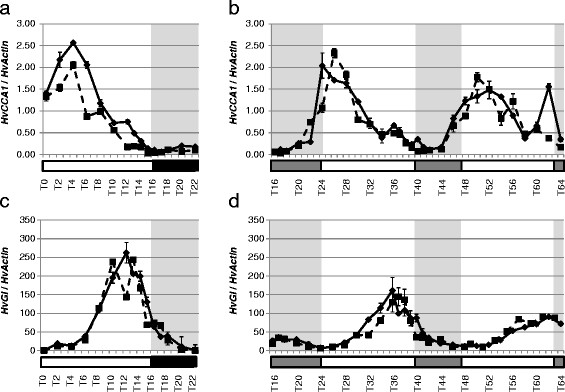
**Diurnal and circadian expression of *HvCCA1 * and *HVGI * in the spring cultivar Scarlett *(ppd-H1 *) and the introgression line S42-IL107 *(Ppd-H1)* under long day and constant light.** Scarlett (solid line) and S42-IL107 (dashed line) were grown under long day conditions (16 hours light - left panel) for two weeks and then released in continuous light (right panel). Transcript accumulation was measured at two hour intervals (one-hour interval at the end of the day and beginning of the night/subjective nights) by qRT-PCR analysis of specific genes and normalized to HvActin. Values represent the average of two technical replicates ± standard error. Black and grey bars indicate objective and subjective nights.

In order to study the functional conservation of transcript accumulation rhythms in the circadian clock of barley, clock orthologs were analyzed for diurnal and circadian expression patterns from young leaf tissue. In addition, we tested the effects of the natural mutation in the CCT domain of *Ppd-H1* (*PRR37*) in the spring barley Scarlett on diurnal and circadian expression of clock and clock output genes. Plants of both genotypes were entrained under long day photoperiods (16-h light/8-h dark) for 2 weeks. Subsequently, leaf samples were harvested every two hours under light/dark (LD) for 24 hours and under continuous light (LL) for 48 hours. Additional sampling (every hour) was performed at the end of the day and beginning of the night (or subjective nights). This provided both a diurnal and free-running sampling series. The expression of circadian-clock orthologs in barley seedlings oscillated under LD, and the rhythm was sustained under LL conditions. The PRR orthologs were expressed in a sequential manner in the order of Hv*PRR7*3/*HvPRR37, HvPRR95/HvPRR59* and *HvPRR1*. Expression of *HvPRR37* (*Ppd-H1*) and Hv*PRR7*3 started rising at T2, *HvPRR95* at T4, *HvPRR59* at T6, and *HvPRR1* at T8. Expression peaks of *HvPRR37* (*Ppd-H1*) and Hv*PRR7*3 were broader than those of the other HvPRR genes, so that expression peaks of *HvPRR37* (*Ppd-H1*) and Hv*PRR7*3 coincided with that of *HvPRR59* and *HvPRR95* at T8, whhile *HvPRR1* peaked at T12 (Figure 2). *HvCCA1* expression started rising before subjective dawn and showed rhythmic peaks 4 hours after light on under LD and 4 hours after subjective dawn under LL (Figures 3a, b). *HvGI* exhibited an expression peak between T10-T14 under LD conditions and T12-T14 under LL conditions (Figures 3c, d). We did not observe significant differences in the expression patterns, period or amplitude of barley circadian-clock genes between the two genotypes differing at the natural mutation in *Ppd-H1*. The sustained oscillations of barley clock genes under free-running conditions showed that these are under circadian control (Figure 2, Figure 3). The natural mutation in the CCT domain of *Ppd-H1* in barley did not affect diurnal or circadian expression of barley clock gene orthologs.

### Diurnal and circadian expression of clock output genes

In Arabidopsis, important circadian-controlled processes include the photoperiod-dependent control of flowering time [46,47]. In order to further characterize the barley circadian clock and test for effects of the natural mutation at *Ppd-H1* on clock output pathways, we analyzed diurnal and circadian expression of candidate genes from the photoperiod pathway. We selected *HvCO1* and *HvCO2*, the barley orthologs of the circadian controlled photoperiod response gene *CONSTANS*[41,48] and *HvFT1*, the barley ortholog of the Arabidopsis *FT*[49].

**Figure 4:**
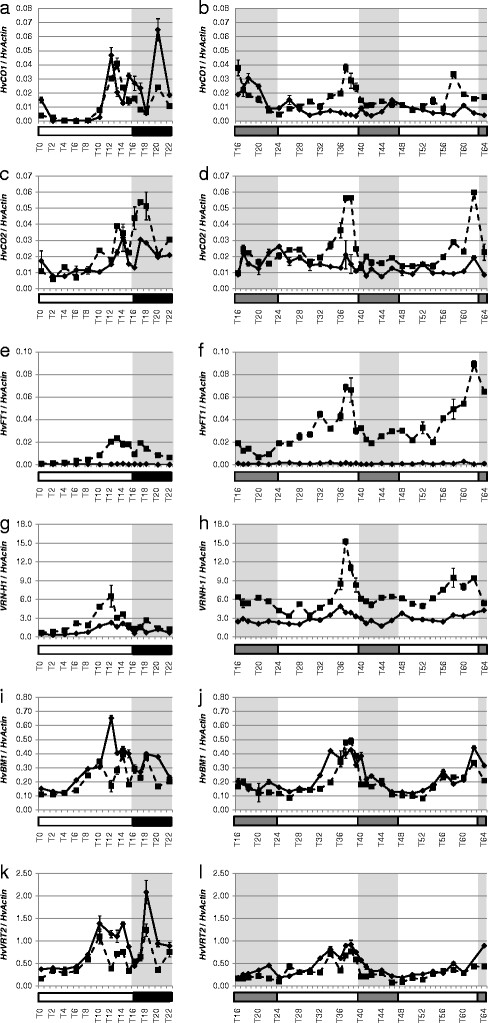
**Diurnal and circadian expression of flowering genes in the spring cultivar Scarlett (*ppd-H1 *) and the introgression line S42 IL-107 (*Ppd-H1 *) under long day and free running conditions.** Scarlett (solid line) and S42-IL107 (dashed line) were grown under long day conditions (16 hours light - left panel) for two weeks and then released in continuous light (right panel). Transcript accumulation was measured at two hour intervals (one-hour interval at the end of the day and beginning of the night/subjective nights) by qRT-PCR analysis of specific genes and normalized to HvActin. Values represent the average of two technical replicates ± standard error. Black and grey bars indicate objective and subjective nights.

In addition, three MADS box transcription factors involved in flowering time control in barley were selected. These include *Vrn-H1*, a flowering activator putatively downstream of *HvFT1*[32], and two repressors of flowering, *HvVRT2* and *HvBM1* orthologous to *SVP* (Short Vegetative Phase) in Arabidopsis [31]. Expression profiles under LD and LL conditions showed that all six genes cycled under diurnal and circadian conditions in at least one genotype (Figure 4). Differences in expression between the two genotypes differing at *Ppd-H1* were observed for *HvCO1, HvCO2, HvFT1,Vrn-H1, HvVRT2,* and *HvBM1*. Under LD conditions, *HvCO1* and *HvCO2* expression cycled in both genotypes, but *HvCO2* expression was lower in Scarlett (*Ppd-H1*) than S42-IL107 (*Ppd-H1*) between T16 and T18 (Figures 4a, c). Under LL, *HvCO1* and *HvCO2* continued to cycle approximately once every 24-h in S42-IL107, while expression peaks of both genes were two- to threefold lower in Scarlett as compared to S42-IL107 (Figures 4b, d). In addition, in Scarlett, *HvCO1* expression peaked at T18 and T46 and showed a strong delay in oscillation peaks under LL. *HvCO2* only displayed a circadian rhythm in S42-IL107 (*Ppd-H1*) and showed an arrhythmic and low expression under LL in Scarlett, which harbors the mutated *Ppd-H1* allele. *HvFT1* expression was below detection in Scarlett, but in S42-IL107, it oscillated with a peak in the afternoon (T13), both under LD and LL (Figures 4e, f). *Vrn-H1* showed a significantly lower expression in Scarlett than in S42-IL107 under LD (Figure 4g). Under LL, *Vrn-H1* expression only cycled in S42-IL107, but not in Scarlett (Figure 4h). *HvVRT2* and *HvBM1* cycled under LD and LL conditions in both genotypes (Figures 4i-l). S42-IL107 (*Ppd-H1*) exhibited a lower expression of *HvVRT2* than Scarlett (*Ppd-H1*) under LD and LL. All analyzed flowering-time genes were thus characterized by rhythmic expression that persisted under LL conditions. However, the natural mutation in *Ppd-H1* strongly delayed and dampened circadian rhythms of *HvCO1, HvCO2* and *Vrn-H1* under LL. Furthermore, *HvFT1* expression could not be detected in Scarlett with the mutated *Ppd-H1* allele.

**Figure 5:**
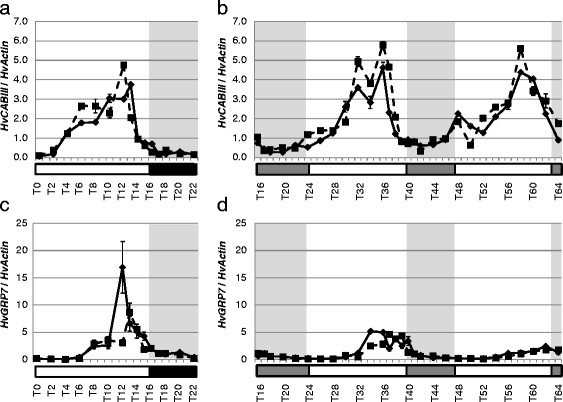
**Diurnal and circadian expression of clock output genes *HvCABII * and *HvGRP7 * in the spring cultivar Scarlett (ppd-H1) and the introgression line S42 IL-107 (*Ppd-H1 *) under long day and free running conditions.** Scarlett (solid line) and S42-IL107 (dashed line) were grown under long day conditions (16 hours light - left panel) for two weeks and then released in continuous light (right panel). Transcript accumulation was measured at two hour intervals (one-hour interval at the end of the day and beginning of the night/subjective nights) by qRT-PCR analysis of specific genes and normalized to HvActin. Values represent the average of two technical replicates ± standard error. Black and grey bars indicate objective and subjective nights.

In addition, we analyzed diurnal and circadian expression of the chlorophyll a/b-binding protein *HvCABIII*[50] and *HvGRP7*[51], the barley ortholog of the Glycine Rich RNA-binding protein *GRP7,* also termed cold-circadian rhythms-RNA binding (*CCR2*) [52]. These two genes are known clock output genes from the photosynthetic pathway [53] and slave (non-self-sustaining) oscillator [51], respectively. We found that both genes showed rhythmic expression under LD and LL and no significant differences were observed between genotypes (Figures 5a–d). These two genes were thus under circadian control in barley and were not affected by variation at *Ppd-H1*.

## Discussion

### The structure and expression profiles of barley clock orthologs are conserved

Early work in wheat has already demonstrated that monocots possess an endogenous circadian clock, *Cab-1* gene expression continued to cycle in wheat plants that had been transferred to continuous light or darkness [54]. The recent availability of monocot plant-genome sequences allows determination of the extent to which the genetic clock model developed from Arabidopsis is conserved and can thus explain endogenous cycling of gene expression in monocot crops. In this work, we demonstrated structural similarities between the potential barley clock orthologs and rice and Arabidopsis clock genes (Figure 1, Additional file 2: Figure S1, Additional file 3: Figure S2, Additional file 4: Figure S3, Additional file 5: Table S2). PRR genes from six different monocot species and Arabidopsis as an eudicot species clearly separated into three clades (PRR1/TOC1 clade, PRR3/7 clade, and PRR5/9 clade) (Figure 1). Each clade contained genes from both eudicots and monocots, confirming that ancient PRR gene(s) diverged into three clades before the speciation of monocots and eudicots [55]. Within the PRR3/7 and PRR5/9 clade the relationships between the Arabidopsis genes and monocot orthologs could not be resolved confirming that since the divergence of monocot and eudicots these clades have expanded independently in both lineages as a result of genome duplication [55]. Additionally, the high average distance of PRR37/73 and PRR59/95 orthologs, as compared to the other clock genes, suggests functional divergence and specialization that occurred in the monocot lineage. In contrast, *CCA1* sequences showed a high similarity within the monocots (0.27), as compared to the two paralogous genes, *CCA1* and LHY in Arabidopsis (0.75). This suggested conservation of *CCA1* gene function in monocots as compared to stronger diversification between the paralogous genes in Arabidopsis (Figure 1b, Table 2, Additional file 5: Table S2). Two *CCA1* paralogs were found in the monocotyledonous plant *Lemna,* suggesting that duplications/deletions of clock genes occurred throughout the evolution of eudicots and monocots [56]. Phylogenetic analysis of GI sequences confirmed high amino-acid sequence identities within the monocots and between monocots and Arabidopsis. High levels of conservation of GI have already been demonstrated for seed plants, including monocotyledonous plants, such as rice (*Oryza sativa*) [57], Brachypodium [58], wheat [59], barley [40], and the eudicotyledons, such as pea [60]. Our data suggest that Arabidopsis clock genes are conserved in barley and other monocot species. However, differences in gene number (*CCA1/LHY*) and genetic differentiation within gene families (PRR gene family) suggest evolutionary modification of clock related components between barley and Arabidopsis.

The similarities between the clock amino-acid sequences of *H. vulgare* and Arabidopsis suggest similar molecular functions of these proteins. Barley clock genes cycled under free-running conditions and their temporal expression profiles were similar to those in Arabidopsis. This indicated that transcriptional regulatory mechanisms are likely conserved between these species. Functional conservation of some clock orthologs has been demonstrated for the monocotyledonous plants rice [21] and *Lemna*[22]. Conservation of expression patterns of barley clock orthologs under LD and LL support the suggestion that clock orthologs function in the circadian clock in barley.

### Natural genetic variation in *Ppd-H1* affects photoperiod output genes

Despite structural and functional conservation, divergence between Arabidopsis and monocot clock genes is suggested by evolving functions in the *PRR7* lineage in monocots. In the monocots, barley and wheat, *PRR37* (*Ppd-H1* and Ppd-A1-D1, respectively) is one of the major determinants of photoperiod sensitivity and flowering time [27,61]. In Arabidopsis, *PRR7* contributes to the determination of flowering time, although the effects are not large and *PRR7* may not be major determinant of flowering time among natural populations [62]. In Arabidopsis expression of the PRR1/TOC1 family members is under the control of a coordinate circadian rhythm at the level of transcription such that the PRRs mRNAs start accumulating sequentially after dawn with 2 to 3 hours intervals in the order of *PRR9, PRR7, PRR5, PRR3,* and *PRR1/TOC1*[63]. It was demonstrated that light stimuli and possibly phytochromes are crucial to induce the early transcription of *PRR9,* and this event appears to link the intrinsic oscillation of the *AtPRR1/TOC1* circadian waves to the entrained external photoperiod [64]. Like in Arabidopsis, *PRR* orthologs in barley were also expressed in a sequential manner at approximately 2 hours intervals from each other (Figure 2). However, in contrast to Arabidopsis *Ppd-H1* (*HvPRR37*) and Hv*PRR7*3, and not the barley orthologs of PRR9, showed the earliest increase in expression after dawn and were followed by expression of *HvPRR95/HvPRR59* and *HvPRR1*. This expression pattern was similar to the sequential expression of *PRRs* in rice with *OsPRR7*3 (*OsPRR37*) induced first after dawn, followed by *OsPRR95* (*OsPRR59*) and *OsPRR1*[23]. In rice, the circadian-shape of *OsPRR7*3 was markedly affected by the photoperiodic conditions, whereas *OsPRR1* was not affected by changes in photoperiod. Such photoperiodicity-dependent changes in the circadian-profiles have been reported for certain circadian controlled Arabidopsis genes, which include the *CO* gene that encodes a crucial regulator of flowering time. These findings suggest differences in the control of PRRs between Arabidopsis and the monocots rice and barley. Differential regulation of PRRs in Arabidopsis and monocots may be the key to understand why natural mutations in *Ppd-H1* (*HvPRR37*) and (*Hd2*) *OsPRR7*3 cause variation in flowering time in barley and rice [27,65], while mutations of *PRR7* do not have strong effects on development in Arabidopsis.

The *PRR7* orthologs in barley and wheat may affect flowering through changing circadian parameters or through a clock independent function of the gene. In order to pursue this question, we tested the effects of a natural mutation at *Ppd-H1* on diurnal and circadian expression profiles of core clock and output genes. Expression of barley clock genes and the clock output genes *HvCABIII* and *HvGRP7* did not differ between the two genotypes harboring a different *Ppd-H1* allele under LD or LL (Figures 2, 3, 5). These results suggest that the mutation in the CCT domain of *Ppd-H1* does not affect diurnal and circadian cycling of clock genes in barley. In contrast, expression profiles of barley flowering-time genes showed significant differences between both genotypes. The *Ppd-H1* mutant was found to be arrhythmic under free-running conditions for the photoperiod-response genes HvCO1, *HvCO2*, and the MADS-box transcription factor and vernalization responsive gene *Vrn-H1* (Figure 4). A wild-type *Ppd-H1* allele was thus necessary to maintain circadian oscillations of these genes under constant light. Cycling of *HvCO1, HvCO2*, and *Vrn-H1* was observed in Scarlett (*Ppd-H1*) under LD as compared to LL. This suggested that light/dark cues were necessary for diurnal oscillations in the presence of a mutated *Ppd-H1* allele. Separate effects of the mutation in *Ppd-H1* on circadian expression of flowering time genes and clock orthologs indicate independent functions of this gene in the clock and photoperiod pathways.

PRR9, *PRR7*, and PRR5 positively regulate flowering time in Arabidopsis through the activation of *CO* expression during daytime [66]. Our results show that under LL, variation at *Ppd-H1* also controlled *HvCO-like* gene expression in barley. However, under LD, variation at *Ppd-H1* did not show clear effects on diurnal expression profiles of *HvCO1* and *HvCO2*; while the mutation in *Ppd-H1* had a significant effect on *HvFT1* expression levels also under LD. *Ppd-H1* may thus also affect *HvFT1* expression independently of *HvCO-like* gene transcript accumulation under LD. These results are supported by a recent analysis of a spring barley Bowman and a derived introgression line carrying a mutation in *HvElf3,* the barley ortholog of *Elf3* in Arabidopsis. Elevated levels of *HvFT1* expression and early flowering in the introgression line as compared to the recurrent parent Bowman did not correlate with higher expression of *HvCO1*[67]. It has already been shown that functional variation at *Ppd-H1* and *Ppd-A1-D1* has a major effect on expression of *HvFT1* and *TaFT* in barley and wheat, respectively [27,61]. Structural conservation of PPD1 in wheat and barley (Figure 1) may thus reflect also functional conservation. However, in contrast to barley, functional variation at *Ppd-D1a* in wheat is associated with a deletion in the promoter and mis-expression of the gene, in contrast to *Ppd-H1* in barley that harbors a change in the protein coding sequence [27,61].

The *PRR37* ortholog of the short day plant Sorghum was recently identified as a repressor of flowering under long day conditions [68]. The repressive function of *SbPRR37* was due to a bimodal expression with peaks in the morning and evening which were controlled by light and the circadian clock. Loss-of-function mutations in *SbPRR37* and short day abolished the evening phase expression of *SbPRR37* and caused significant effects on flowering time and expression of *SbCO* and *SbFT,* the Sorghum orthologs of *CO* and *FT*. Variation at both genes, *Ppd-H1* and *SbPRR37* showed significant effects on expression of the *FT* orthologs and flowering in the respective inductive day-length. In contrast to the study on Sorghum, we show that the floral inducer *Ppd-H1* in the long-day-grass barley does not exhibit the light dependent second expression peak in the evening under LD. In addition, the hypomorphic mutation in *Ppd-H1* caused a reduced and arrhythmic expression of *HvCO1/HvCO2* only under LL, while the loss-of-function allele of *SbPRR37* abolished the second evening peak of *SbCO* under LD and LL, as compared to a bimodal expression of *SbCO* in the Sorghum wild type. Taken together, our results suggest that *PRR37* is regulated differently in long and short day grasses and mutations in the gene have differential effects on downstream genes, photoperiod sensitivity and flowering.

Interestingly, we identified strong correlations in the expression patterns of HvCO1/HvCO2 and *Vrn-H1* under LD and LL. Correlation of expression patterns may indicate that *Vrn-H1* expression is affected by *Ppd-H1* through HvCO1/HvCO2. Expression of *Vrn-H1* in Scarlett was reduced, but cycled in LD, and was dampened with a trend towards arrhythmicity in LL (Figures 4g, h). It has been reported that *Vrn-H1* is primarily controlled by vernalization, while photoperiod affects *Vrn-H1* expression only indirectly through controlling Vrn-H2 expression [32]. Our results indicate that *Vrn-H1* expression is circadian controlled and directly or indirectly affected by the mutation in *Ppd-H1*. In Arabidopsis, the *Vrn-H1* orthologs *AP1* and *FUL* have not been reported to cycle under constant conditions [69]. In barley, *HvVRT2* and *HvBM1,* which act as repressors of flowering in barley [31], were also under circadian control. Both genes showed higher expression levels in the late flowering genotype Scarlett, but diurnal and circadian cycling did not differ significantly between the two genotypes. Lower expression of *HvVRT2* and *HvBM1* in the early flowering S42-IL107 (*Ppd-H1*) than in the late flowering line Scarlett (*Ppd-H1*) may thus reflect differences in development rather than direct effects of the mutation in *Ppd-H1*.

## Conclusion

It was first shown in wheat that transcript accumulation of the *Cab-1* gene, encoding the light-harvesting chlorophyll a/b binding protein, was under circadian control [54]. However, the more tractable model plant Arabidopsis served then to advance the mechanistic understanding of how the circadian clock keeps time in plants. Recent technical advances enable us now to return to the crops and to study inter- and intraspecific modulation of circadian time-keeping and its effects on adaptation and fitness in crops. The present study has demonstrated a high degree of conservation of the circadian clock genes in barley as compared to Arabidopsis and rice. However, differences in gene numbers, diversity, and in the function of *Ppd-H1* suggest evolutionary modification of clock related components. Our results indicate that *Ppd-H1* in barley has evolved novel functions in the control of flowering time, which are independent of its function in the circadian clock. Direct or indirect regulation of *Vrn-H1* by the circadian clock and *Ppd-H1* suggest modification of the photoperiod response pathway in barley as compared to Arabidopsis where the orthologs *AP1* and *FUL* are not known to be under circadian control. In addition, our results suggested differences in the regulation of *PRR37* (*Ppd-H1*) in the long-day grass barley as compared to the short-day grass Sorghum. Structural and functional characterization of the barley circadian clock will set the basis for future studies of the adaptive significance of the clock in *Triticeae* species.

## Authors’ contributions

CC carried out the sequencing, phylogenetic analyses, expression profiling and drafted the manuscript. MS contributed to the expression profiling. SJD conceived the study and drafted the manuscript. MK conceived the study, participated in its design and coordination, and drafted the manuscript. All authors read and approved the final manuscript.

## Supplementary Material

Additional file 1 List of primers used to clone *HvPRR1* and *HvCCA1* and to perform Real Time qRT-PCR. Click here for file

Additional file 2 Protein alignment of GIGANTEA. Circles indicate the region containing four clusters of basic amino-acids (asterisks) that were demonstrated to be sufficient in Arabidopsis for nuclear targeting [70]. Click here for file

Additional file 3 Protein alignment of CCA1/LHY gene family. Asterisks indicate the conserved MYB domain. Click here for file

Additional file 4 Protein alignment of PRR gene family. Asterisks and circles indicate the conserved Pseudo receiver and CCT domains, respectively. Click here for file

Additional file 5 Estimates of average genetic distance between GIGANTEA (a), CCA1/LHY (b) and PRRs (c) sequences. Click here for file
