# Supplementary material for: Expression conservation within the circadian clock of a monocot: natural variation at barley Ppd-H1 affects circadian expression of flowering time genes, but not clock orthologs
Source: BMC Plant Biol. 2012 Jun 21;12:97. doi: 10.1186/1471-2229-12-97 (PMC3478166; doi:10.1186/1471-2229-12-97)
Supplement: Additional file 1 — List of primers used to clone HvPRR1 and HvCCA1 and to perform Real Time qRT-PCR. [file 1471-2229-12-97-S1.pdf]

Supplementary tables

Supplementary Table 1: List of primers used to clone HvPRR1 and HvCCA1 and to perform qRT-PCR

Gene Name	GeneBank#	Primer Name	Primer Sequence	Method
HvCCA1	TC172149, TC159049 (based on)	CCA1_605F(CLO)	GTAGACGCCCTCAACTCCAA	gene cloning
HvCCA1	TC157959, TC154148, DN184424 (based on)	CCA1_2881R(CLO)	CTTGTGCAGGCTGGTAATCA	gene cloning
HvPRR1	TC177049, CA002857 (based on)	PRR1_53F(CLO)	GTTGACCGGAGCAAGGTG	gene cloning
HvPRR1	TC177049, CA002857 (based on)	PRR1_1620R(CLO)	CTATGCTTTCTCAGTTGCAAAGT	gene cloning
HvACTIN	Contig1934_s_at	ACT_591F	CGTGTTGGATTCTGGTGATG	qRT
HvACTIN	Contig1934_s_at	ACT_789R	AGCCACATATGCGAGCTTCT	qRT
HvPRR1	JN603243	HvPRR1-1056F	GAGCATAGCATGGCACTTCA	qRT
HvPRR1	JN603243	HvPRR1-1292R	TGTCTTTCCTCGGAAATTGG	qRT
HvPRR37 (PPD-H1)	AY970701	PPD1_2165F(qRT)	GATGGATTCAAAGGCAAGGA	qRT
HvPRR37 (PPD-H1)	AY970701	PPD1_2336R(qRT)	GAACAATTGGCTCCTCCAAA	qRT
HvPRR73	AK376549	PRR73_441F(qRT)	GCGCCGTAGAGAATCAGAAC	qRT
HvPRR73	AK376549	PRR73_662R(qRT)	CATGTCGGGTACAGTCATCG	qRT
HvPRR59	AK361360	PRR59_2064F(qRT)	GAAATTCCGCATGAAAAGGA	qRT
HvPRR59	AK361360	PRR59_2212R(qRT)	TTCCGCATCTTCTGTTGTTG	qRT
HvPRR95	AK252005	PRR95_1467F(qRT)	CAGAACTCCAGTGTCGCAAA	qRT
HvPRR95	AK252005	PRR95_1717R(qRT)	TGCTGTTGCCAGAGTTGTTC	qRT
HvCCA1	JN603242	HvCCA1-672F	CCTGGAATTGGAGATGGAGA	qRT
HvCCA1	JN603242	HvCCA1-882R	TGAGCATGGCTTCTGATTTG	qRT
HvGI	AY740524	GI_6780F	TCAGTTAGAGCTCCTGGAAGT	qRT
HvGI	AY740524	GI_7289R	GGTAGTTTGGGCTTTGGATG	qRT
HvCO1	AF490468	CO1_2185F(qRT)	CTGCTGGGGCTAGTGCTTAC	qRT
HvCO1	AF490468	CO1_3454R(qRT)	CCTTGTTGCATAACGTGTGG	qRT
HvCO2	AF490470	CO2_564F(qRT)	AGTGGACTCTTGGCTCCTCA	qRT
HvCO2	AF490470	CO2_721R(qRT)	CATGCTGCTGTTCTTGCATT	qRT
HvFT1/VRN3	DQ100327	FT1_1955F	GGTAGACCCAGATGCTCCAA	qRT
HvFT1/VRN3	DQ100327	FT1_2183R	CAGGAGGTGATGTGCTACGA	qRT
VRN-H1	AY750995	HvBM5A_292F	CTGAAGGCGAAGGTTGAGAC	qRT
VRN-H1	AY750995	HvBM5A_494R	TTCTCCTCCTGCAGTGACCT	qRT
HvVRT2	DQ201168	VRT2_559F	CCGATGTTGTCCCTGAAGAT	qRT
HvVRT2	DQ201168	VRT2_747R	GGAACTCCCTCATGGACTCA	qRT
HvBM1	AJ249141	BM1_F	AGAGGAGAACGCAAGGCTAAAGG	qRT
HvBM1	AJ249141	BM1_R	AGTTGAAGAGTGATAATCCGAGCC	qRT
HvCCR2	AK248251	CCR2_542F(qRT)	ACTGGAGGGAGTGAATGGTG	qRT
HvCCR2	AK248251	CCR2_716R(qRT)	GGAACGGTAGCGTCACATCT	qRT
HvLHCII-typeIII-CAB	X63197	LHCB_454F	TCTGAGGGTGGTCTCGATTA	qRT
HvLHCII-typeIII-CAB	X63197	LHCB_550R	CAACAAGACCCATGAGAAGG	qRT
