# Supplementary material for: Expression conservation within the circadian clock of a monocot: natural variation at barley Ppd-H1 affects circadian expression of flowering time genes, but not clock orthologs
Source: BMC Plant Biol. 2012 Jun 21;12:97. doi: 10.1186/1471-2229-12-97 (PMC3478166; doi:10.1186/1471-2229-12-97)
Supplement: Additional file 2 — Protein alignment of GIGANTEA. Circles indicate the region containing four clusters of basic amino-acids (asterisks) that were demonstrated to be sufficient in Arabidopsis for nuclear targeting [70]. [file 1471-2229-12-97-S2.pdf]

```
                10         20         30         40         50         60         70
        ....|....| ....|....| ....|....| ....|....| ....|....| ....|....| ....|....|
HvGI    --MSASNGKW IDGLQFSSLF WPPPHDAQQK QAQILAYVEY FGQFTSDSEQ FPEDVAQLIQ TCYPSKEKRL
TaGI    --MSVSNGKW IDGLQFSSLF WPPPHDVQQK QAQILAYVEY FGQFTSDSEQ FPEDVAQLIQ SCYPSKEKRL
BdGI    --MSASNGKW IDGLQYSSLF WPPPHDAQQK QVQILAYVEY FGQFTSDSEQ FPEDVAQLIQ SCYPAKEKRL
OsGI    --MSASNEKW IDGLQFSSLF WPPPQDSQQK QAQILAYVEY FGQFTADSEQ FPEDIAQLIQ SCYPSKEKRL
ZmGI    --MSDSNVKW IDGLQFTSLY WPPPLDAEQK QAQILAYVEY FGQFTADTDQ FPEDIAQLIQ SSYPSKENRL
SbGI    --MSDSNVKW IDGLQFTSLY WPPPQDVEQK QAQILAYVEY FGQFTADSEQ FPEDVAQLIQ SSYPSKESRL
AtGI    MASSSSSERW IDGLQFSSLL WPPPRDPQQH KDQVVAYVEY FGQFTS--EQ FPDDIAELVR HQYPSTEKRL

                80         90        100        110        120        130        140
        ....|....| ....|....| ....|....| ....|....| ....|....| ....|....| ....|....|
HvGI    VDEVLATFVL HHPEHGHAVV HPILSRIIDG TLSYDSHGSP FNSFISLFTQ SSEKEYSEQW ALACGEILRV
TaGI    VDEVLATFVL HHPEHGHAVV HPILSRIIDG TLSYDSHGSP FNSFISLFTQ SSEKEYSEQW ALACGEILRV
BdGI    VDEVLATFVL HHPEHGHAVV HPILSRIIDG TLSYDRHGFP FNSFISLFTQ TSEKEYSEQW ALACGEILRV
OsGI    VDEVLATFVL HHPEHGHAVV HPILSRIIDG TLSYDRNGFP FMSFISLFSH TSEKEYSEQW ALACGEILRV
ZmGI    VDEVLATFVL HHPEHGHAVA HPILSRIIDG TLCYDRHGPP FSSFISLFSH NSEQEYSEQW ALACGEILRV
SbGI    VDEVLATFVL HHPEHGHAVV HPILSRIIDG TLCYDRHGPP FSSFISLFSH TSEQEYSEQW ALACGEILRV
AtGI    LDDVLAMFVL HHPEHGHAVI LPIISCLIDG SLVYSKEAHP FASFISLVCP SSENDYSEQW ALACGEILRI

               150        160        170        180        190        200        210
        ....|....| ....|....| ....|....| ....|....| ....|....| ....|....| ....|....|
HvGI    LTHYNRPIFK V------ADC NNTSDQATTS CSAQEKANYS PGNEPERKPL RPLSPWITDI LLTAPLGIRS
TaGI    LTHYNRPIFK V------ADC NHQIRPGHSK LFCTEKAITL PGNEPEGKPL RPLSPWITDI VLTAPLGIRS
BdGI    LTHYNRPIFK V------AER NSTSDQATAS YSVQEKANGS PGNEPDRKPL RPLSPWITDI LLTAPLGIRS
OsGI    LTHYNRPIFK VDHQHSEAEC SSTSDQASSC ESMEKRANGS PRNEPDRKPL RPLSPWITDI LLAAPLGIRS
ZmGI    LTHYNRPIFK VERQHTEAEC SSTSDQATSS DSTDKRSNNS PGNESDWKPL RPLTPWITDI LLAAPLGIRS
SbGI    LTHYNRPIFK VERQHSEAEC SSTSDQATSS DSTDKKSNNS PGNESDRKPL RPLTPWITDI LLAAPLGIRS
AtGI    LTHYNRPIYK TEQQNGDTER NCLSKATTSG SPTSEPKAGS P-TQHERKPL RPLSPWISDI LLAAPLGIRS

               220        230        240        250        260        270        280
        ....|....| ....|....| ....|....| ....|....| ....|....| ....|....| ....|....|
HvGI    DYFRWCGGVM GKYAAGGELK PPTTAYSRGA GKHPQLMPST PRWAVANGAG VILSVCDEEV ARYETANLTA
TaGI    DYFRWCGGVM GKYAAGGELK PPTTAYSRGA GKHPQLMPST PRWAVANGAG VILSVCDEEV ARYETANLTA
BdGI    DYFRWCGGVM GKYAAGGELK PPTTAYSRGA GKHPQLMPST PRWAVANGAG VILSVCDEEV ARYETANLTA
OsGI    DYFRWCGGVM GKYAAGGELK PPTTAYSRGS GKHPQLMPST PRWAVANGAG VILSVCDEEV ARYETANLTA
ZmGI    DYFRWCGGVM GKYAAGGELK PPTTACSRGS GKHPQLMPST PRWAVANGAG VILSVCDEEV ARYETANLTA
SbGI    DYFRWCGGVM GKYAAGGELK PPTTACSRGS GKHPQLMPST PRWAVANGAG VILSVCDEEV ARYETANLTA
AtGI    DYFRWCSGVM GKYAAG-ELK PPTIAS-RGS GKHPQLMPST PRWAVANGAG VILSVCDDEV ARYETATLTA

               290        300        310        320        330        340        350
        ....|....| ....|....| ....|....| ....|....| ....|....| ....|....| ....|....|
HvGI    AAVPALLLPP PTTPLDEHLV AGLPPLEPYA RLFHRYYAIA TPSATQRLLF GLLEAPPSWA PDALDAAVQL
TaGI    AAVPALLLPP PTTPLDEHLV AGLPPLEPYA RLFHRYYAIA TPSATQRLLF GLLEAPPSWA PDALDAAVQL
BdGI    AAVPALLLPP PTTPLDEHLV AGLPPLEPYA RLFHRYYAIA TPSATQRLLF GLLEAPPSWA PDALDAAVQL
OsGI    AAVPALLLPP PTTPLDEHLV AGLPPLEPYA RLFHRYYAIA TPSATQRLLF GLLEAPPSWA PDALDAAVQL
ZmGI    AAVPALLLPP PTTPLDEHLV AGLPPLEPYA RLFHRYYAIA TPSATQRLLF GLLEAPPSWA PDALDAAVQL
SbGI    AAVPALLLPP PTTPLDEHLV AGLPPLEPYA RLFHRYYAIA TPSATQRLLF GLLEAPPSWA PDALDAAVQL
AtGI    VAVPALLLPP PTTSLDEHLV AGLPALEPYA RLFHRYYAIA TPSATQRLLL GLLEAPPSWA PDALDAAVQL

               360        370        380        390        400        410        420
        ....|....| ....|....| ....|....| ....|....| ....|....| ....|....| ....|....|
HvGI    VELLRAAEDY ATGMRLPKNW LHLHFLRAIG TAMSMRAGIA ADTAAALLFR ILSQPTLLFP PLRHAEGVEV
TaGI    VELLRAAEDY ATGMRLPKNW LHLHFLRAIG TAMSMRAGIA ADTAAALLFR ILSQPMLLFP PLRHAEGVEV
BdGI    VELLRAAEDY ATGMRLPKNW LHLHFLRAIG TAMSMRAGMA ADTAAALLFR ILSQPTLLFP PLRHAEGLEV
OsGI    VELLRAAEDY DSGMRLPKNW MHLHFLRAIG TAMSMRAGIA ADTSAALLFR ILSQPTLLFP PLRHAEGVEL
ZmGI    VELLRAAEDY ASGMRLPKNW MHLHFLRAIG TAMSMRAGIA ADTAAALLFR ILSQPTLLFP PLRHAEGVEV
SbGI    VELLRAAEDY ASGMRLPKNW MHLHFLRAIG TAMSMRAGIA ADTAAALLFR ILSQPTLLFP PLRHAEGVEV
AtGI    VELLRAAEDY ASGVRLPRNW MHLHFLRAIG IAMSMRAGVA ADAAAALLFR ILSQPALLFP PLSQVEGVEI

               430        440        450        460        470        480        490
        ....|....| ....|....| ....|....| ....|....| ....|....| ....|....| ....|....|
HvGI    QHEPLGGYVS SYKRQLEVPA SETTIDATAQ GIASLLCAHG PDVEWRICTI WEAAYGLLPL NSSAVDLPEI
TaGI    QHEPLGGYVS SYKRQLEVPA SETTIDATAQ GIASLLCAHG PDVEWRICTI WEAAYGLLPL NSSAVDLPEI
BdGI    QHEPLGGYVS SYKRQLEVPA SETTIDATAQ GIASLLCAHG PDVEWRICTI WEAAYGLLPL NSSAVDLPEI
OsGI    HHEPLGGYVS SYKRQLEVPA SEATIDATAQ GIASMLCAHG PDVEWRICTI WEAAYGLLPL SSSAVDLPEI
ZmGI    HHEPLGGYVS SYKKQLEVPA SEATIDATAQ GIASLLCAHG PDVEWRICTI WEAAYGLLPL SSSAVDLPEI
SbGI    HHEPLGGYVS SYKKQLEVPA SEATIDATAQ GIASLLCAHG PDVEWRICTI WEAAYGLLPL SSSAVDLPEI
AtGI    QHAPIGGYSS NYRKQIEVPA AEATIEATAQ GIASMLCAHG PEVEWRICTI WEAAYGLIPL NSSAVDLPEI

               500        510        520        530        540        550        560
        ....|....| ....|....| ....|....| ....|....| ....|....| ....|....| ....|....|
HvGI    VVAAPLQPPT LSWSLYLPLL KVFEYLPRGS PSEACLMRIF VATVEAILRR TFPSETS-ES SKRP------
TaGI    VVAAPLQPPT LSWSLYLPLL KVFEYLPRGS PSEACLMRIF VATVEAILRR TFPSETS-ES SKRP------
BdGI    VVAAPLQPPT LSWSLYLPLL KVFEYLPRGS PSEACLMRIF VATVEAILRR TFPSETS-EP SRKP------
OsGI    VVAAPLQPPT LSWSLYLPLL KVFEYLPRGS PSEACLMRIF VATVEAILRR TFPSETS-EQ SRKP------
ZmGI    VVAAPLQPPT LSWSLYLPLL KVFEYLPRGS PSEACLMRIF VATVEAILRR TFPSETS-EQ SRKP------
```

```
SbGI VVAAPLQPPT LSWSLYLPLL KVFEYLPRGS PSEACLMRIF VATVEAILRR AFPSETS-EQ SRKP------
AtGI IVATPLQPPI LSWNLYIPLL KVLEYLPRGS PSEACLMKIF VATVETILSR TFPPESSREL TRKARSSFTT

                    570        580        590        600        610        620        630
             ....|....| ....|....| ....|....| ....|....| ....|....| ....|....| ....|....|
HvGI RSQSKNLAVA ELRTMIHSLF VESCASMNLA SRLLFVVLTV CVSHQALPGG SKRP-----T GSENHSSEEA
TaGI RSQSKNLAVA ELRTMIHSLF VESCASMNLA SRLLFVVLTV CVSHQALPGG SKRP-----T GSENHSSEEA
BdGI RSQSKNLAVA ELRTMIHSLF VESCASMNLA SRLLFVVLTV CVSHQALPGG SKRP-----T GSDNHSSEEA
OsGI RSQSKNLAVA ELRTMIHSLF VESCASMDLA SRLLFVVLTV CVSHQALPGG SKRP-----T GSDNHSSEEV
ZmGI RSQSKNLAVA ELHTMIHSLF VESCASMDLA SRLLFVVLTV CVSHQALPGG SKRP-----T GSDNHSHEEA
SbGI RSQSKNLAVA ELHTMIHSLF VESCASMDLA SRLLFVVLTV CVSHQALPGG SKRP-----T GSDNHSHEEA
AtGI RSATKNLAMS ELRAMVHALF LESCAGVELA SRLLFVVLTV CVSHEAQSSG SKRPRSEYAS TTENIEANQP

                    640        650        660        670        680        690        700
             ....|....| ....|....| ....|....| ....|....| ....|....| ....|....| ....|....|
HvGI TEDPRLTNGR NKVKKKQGPV GTFDSYVLAA VCALSCELQL FPILCKSATN SKVKDSIKIL KPGKNNGISN
TaGI TEDPRLTNGR NRVKKKQGPV GTFDSYVLAA VCALSCELQL FPILCKSATN SNVKDSIKIL KPGKNNGISN
BdGI TEGSRLTNGR NRVKKKQGPV GTFDSYVLAA VCALSCELQL FPILCKNGTH SNIKDSIKII MPGKTNGISN
OsGI TNDSRLTNGR NRCKKRQGPV ATFDSYVLAA VCALSCELQL FPFISKNGNH SNLKDSIKIV IPGKTTGISN
ZmGI TEHSRLTNGR SRCKKRQGPV ATFDSYVLAA VCALSCELQL FPFITKNGSH SNLKDSMKII ISGKNNGMNN
SbGI TEHSRLTNGR SRCKKRQGPV ATFDSYVLAA VVALSCELDL FPCISKNGSH SNLKDSLKII IPGKNNGINN
AtGI VSNNQTANRK SRNVKGQGPV AAFDSYVLAA VCALACEVQL YPMISGGGNF SNSAVAGTIT KPVKINGSSK

                    710        720        730        740        750        760        770
             ....|....| ....|....| ....|....| ....|....| ....|....| ....|....| ....|....|
HvGI ELQNSISSAI LHTRRILGIL EALFSLKPSS VGTSWNYSSN EIVAAAMVAA HVSELFRRSR PCLNALSSLK
TaGI ELQNSISSAI LHTRRILGIL EALFSLKPSS VGTSWNYSSN EIVAAAMVAA HASELFRRSK ACLNALSSLK
BdGI ELQNSISSAV IHTRRILGIL EALFSLKPSS VGTSWSYSSN EIVAAAMVAA HVSELFRRSR PCLNALSSLK
OsGI ELHNSISSAI LHTRRILGIL EALFSLKPSS VGTSWSYSSN EIVAAAMVAA HVSELFRRSR PCLNALSALK
ZmGI ELHNSISSAI LHTRRILGIL EAVFSLKPSS VGTSWSYSSN EIVAAAMVAA HVSELFRRSR PCLNALSALM
SbGI EPRSSISSAI LHTRRILGIL EALFSLKPSS VGTSWSYSSN EIVAAAMVAA HVSELFRRSR PCLTSLSAMM
AtGI EYGAGIDSAI SHTRRILAIL EALFSLKPSS VGTPWSYSSS EIVAAAMVAA HISELFRRSK ALTHALSGLM

                    780        790        800        810        820        830        840
             ....|....| ....|....| ....|....| ....|....| ....|....| ....|....| ....|....|
HvGI RCKWDAEIST RASSLYHLID LHGKTVSSIV NKAEPLEAHL TFTSVKRDGQ QHIEENSTSS SGNGNLEKKN
TaGI RCKWDAEIST RASSLYHLID LHGKTVSSIV NKAEPLEAHL TFTSVKRDDE QHIEENGTSS SGSGNLEKKN
BdGI RCKWDAEIST RASSLYHLID LHGKTVSSIV NKAEPLEAHL TLTSVRKDDG QHIEENNVSS SDSDDLGKKN
OsGI QCKWDAEIST RASSLYHLID LHGKTVTSIV NKAEPLEAHL TLTPVKKD-E PPIEEKNINS SDGGALEKKD
ZmGI RCKWDAEIST RASSLYHLID LHGKTVSSIV NKAEPLEAHL TLTPVKRDNQ HHREESNTSS LDSVKLENKN
SbGI RCKRDAEIST RASSLYHLID LHGKTVSSIV NKAEPLEAHL TLTPVKKDNQ HRCEENNTSS SDSVKLENKN
AtGI RCKWDKEIHK RASSLYNLID VHSKVVASIV DKAEPLEAYL KNTPVQKDSV TCLNWKQENT CASTTCFDTA

                    850        860        870        880        890        900        910
             ....|....| ....|....| ....|....| ....|....| ....|....| ....|....| ....|....|
HvGI ASASHMKNGF SRPLLKCSEE ARRNGNVAST SGKVPATLQA EASDLANFLT MDRNGG-YRG SQTLLSSVIS
TaGI GSASHMKNGL SRPLLKCSEE ARRNGNVAST SGKVPATLQA EASDLANFLT MDRNGG-YRG SQTLLRSVIS
BdGI VLASHKKNGF SRPLLNCGEE AIRNGGVAST SGKVIATLQV EASDLANFLT MDRNGG-YGG SQTLLRSVMS
OsGI ASRSHRKNGF ARPLLKCAED VILNGDVAST SGKAIASLQV EASDLANFLT MDRNGG-YRG SQTLLRSVLS
ZmGI GSTSHKKNGF SRPLLKCAEE VLLNGDVAST SGKSIASLQV EASDLANFLT MDRNGG-YRG SQTLLRSVLS
SbGI GSTSHKKNGF SRPLLKCAEE VLLNGDVAST SGKSIASLQV EASDLANFLT MDRNGG-YRG SQTLLRSVLS
AtGI VTSASRTEMN PRGNHKYARH S--DEGSGRP SEKGIKDFLL DASDLANFLT ADRLAGFYCG TQKLLRSVLA

                    920        930        940        950        960        970        980
             ....|....| ....|....| ....|....| ....|....| ....|....| ....|....| ....|....|
HvGI EKQELCFSVV SLLWHKLIAS PETQMSAEST SAHQGWRKVV DALCDVVSAS PAKASTAIVL QAEKDLQPWI
TaGI EKQELSFSVV SLLWHKLIAS PETQMSAEST SAHQGWRKVV DALCDVVSAS PAKASTAIVL QAEKDLQPWI
BdGI EKQELCFSVV SLLWQKLIAS PEMQMSAEST SAHQGWRKVV DALCDVVSAS PAKASTAIVL QAEKDLQPWI
OsGI EKQELCFSVV SLLWQKLIAS PEMQMSAEST SAHQGWRKVV DALCDIVSAS PTKASAAIVL QAEKDLQPWI
ZmGI EKQELCFSVV SLLWQKLIAS PEMQMSAEST SAHQGWRKVV DALCDVVSAS PTKASAAIVL QAEKDLQPWI
SbGI EKQELCFSVV SLLWQKLIAS PEMQMSAEST SAHQGWRKVV DALCDVVSAS PTKASTAIVL QADKDLQPWI
AtGI EKPELSFSVV SLLWHKLIAA PEIQPTAEST SAQQGWRQVV DALCNVVSAT PAKAAAAVVL QAERELQPWI

                    990       1000       1010       1020       1030       1040       1050
             ....|....| ....|....| ....|....| ....|....| ....|....| ....|....| ....|....|
HvGI ARDDEEGQKM WRVNQRIVKL IAELMRNHDS PEALIILASA SDLLLRATDG MLVDGEACTL PQLELLEVTA
TaGI ARDDEEGQKM WRVNQRIVKL IAELMRNHDS PEALIILASA SDLLLRATDG MLVDGEACTL PQLELLEVTA
BdGI ARDDEQGQKM WRVNQRIVKL IAELMRNHDS PEALVILASA SDLLLRATDG MLVDGEACTL PQLELLEVTA
OsGI ARDDEQGQKM WRVNQRIVKL IAELMRNHDS PEALVILASA SDLLLRATDG MLVDGEACTL PQLELLEVTA
ZmGI ARDDEQGQKM WRVNQRIVKL IAELMRNHDS PEALVILASA SDLLLRATDG MLVDGEACTL PQLELLEVTA
SbGI ARDDEQGQKM WRVNQRIVKL IAELMRNHDS PEALVILASA SDLLLRATDG MLVDGEACTL PQLELLEVTA
AtGI AKDDEEGQKM WKINQRIVKV LVELMRNHDR PESLVILASA SDLLLRATDG MLVDGEACTL PQLELLEATA

                   1060       1070       1080       1090       1100       1110       1120
             ....|....| ....|....| ....|....| ....|....| ....|....| ....|....| ....|....|
HvGI RAIHLIVEWG DPGVAVADGL SNLLKCRLSP TIRCLSHASA HVRALSMSVL RDILNSG--P LGSSKTIQG-
TaGI RAIHLIVEWG DPGVAVADGL SNLLKCRLSP TIRCLSHASA HVRALSMSVL RDILNSG--P LGSTKIIQG-
```

```
BdGI  RAIHLIVEWG DPGVAVADGL SNLLKCRLST TIRCLSHPSA HVRALSMSVL RDILNSG--P INSTKIIQG-
OsGI  RAVHLIVEWG DSGVSVADGL SNLLKCRLST TIRCLSHPSA HVRALSMSVL RDILNSG--Q INSSKLIQG-
ZmGI  RAVHLIIEWG DSGLSVADGL SNLLKCRLST TIRCLSHPSA HVRALSMSVL RDILSNG--S VNPNKTIQGE
SbGI  RAVHLIIEWG DSGLSVADGL CNLLKCRLST TIRCLSHPSA HVRALSMSVL RDILNNG--S MNPSKIIQGE
AtGI  RAIQPVLAWG PSGLAVVDGL SNLLKCRLPA TIRCLSHPSA HVRALSTSVL RDIMNQSSIP IKVTPKLPT-

              1130       1140       1150       1160       1170       1180
      ....|....| ....|....| ....|....| ....|....| ....|....| ....|....| .
HvGI  EQRNGIQSPN YQCAAANTVN WQADVERCID WEARSRRATG MTLAFLTAAA NELGCPLPC- -
TaGI  EQRNGIQSPT YQCAAANTVN WQADVERCID WEARSRRATG MTLAFLTAAA NELGCPLPC- -
BdGI  EQRNGVQSPT YQCAAASMVN WQADMERCIE WEARSRRATG MTLAFLSAAA NELGCPLPC- -
OsGI  EHRNGIQSPT YQCLAASIIN WQADVERCIE WEAHSRRATG LTLAFLTAAA KELGCPLTC- -
ZmGI  QQRNGIQSPS YRCLAAGIIN WQADVERCIE WEAHSRRATG LTLAFLSAAA KELGCPLPS- -
SbGI  QQRNGIQNPS YRCLAAGIIN WQADVERCIE WEAHSRRATG LTLAFLSAAA KELGCPLPC- -
AtGI  TEKNGMNSPS YRFFNAASID WKADIQNCLN WEAHSLLSTT MPTQFLDTAA RELGCTISLS Q
```
