# Supplementary material for: Expression conservation within the circadian clock of a monocot: natural variation at barley Ppd-H1 affects circadian expression of flowering time genes, but not clock orthologs
Source: BMC Plant Biol. 2012 Jun 21;12:97. doi: 10.1186/1471-2229-12-97 (PMC3478166; doi:10.1186/1471-2229-12-97)
Supplement: Additional file 3 — Protein alignment of CCA1/LHY gene family. Asterisks indicate the conserved MYB domain. [file 1471-2229-12-97-S3.pdf]

```
                10         20         30         40         50         60         70
        ....|....| ....|....| ....|....| ....|....| ....|....| ....|....| ....|....|
                              **************************************************
HvCCA1  MEINSSGEET VIKVRKPYTI TKQRERWTEA EHKRFLEALK LYGRAWQRIE EHVGTKTAVQ IRSHAQKFFT
TaCCA1  MEINSSGEET VIKVRKPYTI TKQRERWTEA EHKRFLEALK LYGRAWQRIE EHVGTKTAVQ IRSHAQKFFT
BdCCA1  MEMNSSGEET VIKVRKPYTI TKQRERWTEA EHKRFLEALK LYGRAWQRIE EHVGTKTAVQ IRSHAQKFFT
OsCCA1  MEINSSGEEA VVKVRKPYTI TKQRERWTEA EHNRFLEALK LYGRAWQRIE EHVGTKTAVQ IRSHAQKFFT
ZmCCA1  MEVNSSGEET VIKVRKPYTI TKQRERWTEA EHKRFLEALK LYGRAWQRIE EHVGTKTAVQ IRSHAQKFFT
SbCCA1  MEVNSSGEEM VVKVRKPYTI TKQRERWTEA EHKRFLEALK LYGRAWQRIE EHVGTKTAVQ IRSHAQKFFT
AtCCA1  METNSSGEDL VIKTRKPYTI TKQRERWTEE EHNRFIEALR LYGRAWQKIE EHVATKTAVQ IRSHAQKFFS
AtLHY   MDTNTSGEEL LAKARKPYTI TKQRERWTED EHERFLEALR LYGRAWQRIE EHIGTKTAVQ IRSHAQKFFT

                80         90        100        110        120        130        140
        ....|....| ....|....| ....|....| ....|....| ....|....| ....|....| ....|....|
HvCCA1  KLEKEAINNG TSPGQAHDID IPPPRPKRKP NCPYPRKGCL SSETPTREVP -----KSSVS LSNSNSQMES
TaCCA1  KLEKEAINNG TSPGQAHDID IPPPRPKRKP NCPYPRKGCL SSETPTREVP -----KSSVS LSNSNAEMGS
BdCCA1  KLEKEAINNG TSPGEAHDID IPPPRPKRKP NCPYPRKGCL SSDAPTREAP NDKSTKSNIS LTSSNAQTAG
OsCCA1  KLEKEAINNG TSPGQAHDID IPPPRPKRKP NSPYPRKSCL SSETSTREVQ NDKATISNMT N-NSTAQMAG
ZmCCA1  KLEKEAINNG TSPGQAHDID IPPPRPKRKA NSPYPRKSGL SSETPTKELP NSKSTKPNMP LSNENVLMAG
SbCCA1  KLEKEAMTNG TSPGQALDID IPPPRPKRKP NNPYPRKSGL SSETPTKDFP NDKSAKPNMP LSNGNVQMAG
AtCCA1  KVEKEAEAKG VAMGQALDIA IPPPRPKRKP NNPYPRKTGS GTIL------ -----MSKTG VNDGKESLGS
AtLHY   KLEKEAEVKG IPVCQALDIE IPPPRPKRKP NTPYPRKPGN NGTS------ -----SSQVS SAKDAKLVSS

               150        160        170        180        190        200        210
        ....|....| ....|....| ....|....| ....|....| ....|....| ....|....| ....|....|
HvCCA1  NGTLQVTSTQ KLQRKELSGN GSCSEVINIF REAPSASFSS SNKSSSNHGV SGGIEPTKTE IKDMAAMERK
TaCCA1  NGTLQLTCIQ KLQRKELSEN GSCSEVINIF REAPSASFSS SNKSSSNHGV SGGIEPTKTE NKDIATMERK
BdCCA1  N-----ATLQ KLQGKESS-- -SCSEVLNLF RDAQSASFSS VNKSSSNHGA SMEIEATKTE IKDMTTMDRT
OsCCA1  D-----AALE KLQRKEISEK GSCSEVLNLF REVPSASFSS VNKSSSNHGA SRGLEPTKTE VKDVVILERD
ZmCCA1  D-----ASLQ KFQRKKLSGK ESCSEVLNLF RDAPSPSFSS VNKSSSNHGA P--IEASKTK IRDMTIMENS
SbCCA1  D-----ASLQ KFQRKELSEK GSCSEVLNLF RDAPSASFSS VNKSSSNHGA PRGVEASKTE IRDMTIMENN
AtCCA1  E--------- ---------- ---------K VSHPEMANED RQQSKPEEKT -----LQEDN CSDCFTHQYL
AtLHY   A--------- ---------- ---------S SSQLNQAFLD LEKMPFSEKT STGKENQDEN CSGVSTVNKY

               220        230        240        250        260        270        280
        ....|....| ....|....| ....|....| ....|....| ....|....| ....|....| ....|....|
HvCCA1  STSVDVAKDV KDINDQEMER NNRVHISSKY DRSHEDCLDS SMKHMQLKPN TVETTYTGQH VASAPLYQMN
TaCCA1  STSIDVGKDV KDINDQEMER NNRVHISSNY DRSHEDCLDN SMKHMQLKPN TAETTYTGQH AASAPLYQMN
BdCCA1  SVSIDMEKDV KDINDQEMER PNRIHISAKY DHLHDDCLDN SRQHVKLKPK SVEITHTDQH PTRASHYQMD
OsCCA1  SISNGAGKDA KDINDQEMER LNGIHISSKP DHSHENCLDT SSQQFKPKSN SVETTYVDWS AAKASHYQMD
ZmCCA1  SLNPNMQEDV KEINDQEMER LNGTQISSKC EHSHEGYLDI SMQQMKLKPE SVETTDVDKQ TARASHSLAE
SbCCA1  SLNPNMQEDV KEISDHEMER LNSIQISSKC EHSHEGYLDL SMQQMKLKPK SVETTYVDKQ TARASHSLAE
AtCCA1  SAASSMNKSC IETSNASTFR ---EFLPSR- ---EEGSQNN RVRKESNS-- DLNAKSLENG NEQGPQTYP-
AtLHY   PLPTKVSG-D IETSKTSTVD NAVQDVPKKN -KDKDGNDGT TVHSMQNYPW HFHADIVNGN IAKCPQNHPS

               290        300        310        320        330        340        350
        ....|....| ....|....| ....|....| ....|....| ....|....| ....|....| ....|....|
HvCCA1  KTGATGTPDP GTEGSHPDQT NDQVGGANGS MD-CIHPTLP VDLKFGSSST AQPFPHNYSG FAPTMQCQCN
TaCCA1  KTGATGAPDP GTEGSHPDQT SDRVGGANGS MD-CIHPTLP VDPKIGSSST AQSFPHNYAG FAPTMQCHCN
BdCCA1  RTGASGIPVI GTEKSPADQT NDQVGVAKGN MN-CLHPTLS VDPKFDNSST AQPITHNYAA FAPMMQCHCN
OsCCA1  RNGVTGFQAT GTEGSHPDQT SDQMGGASGT MNQCIHPTLP VDPKFDGNAA AQPFPHNYAA FAPMMQCHCN
ZmCCA1  ITGTTSIPVT ATEETHSVLT SDQVG-INGS MNPSIHPMFP ADPKFDSSAT PQPFPHNYAA FAPMMQCNCN
SbCCA1  KNGTASILVT ATEGTHSDQT SDQVG-INGS INPCIHPMLS SDPKFDSSAT PQHFPHNYAA FAPMMQGNCN
AtCCA1  ---------- ---------- ---------- ----MHIPVL VPLG-----S SITSSLSHPP SEPDSHPHTV
AtLHY   GMVSQDF--- ---------- ---------- ----MFHPMR EETHGHANLQ ATTASATTTA SHQAFPACHS

               360        370        380        390        400        410        420
        ....|....| ....|....| ....|....| ....|....| ....|....| ....|....| ....|....|
HvCCA1  QDAYRSSVDM SSTFSNMLVS TLLSNPTVHA AARLAASYWP AADSNIPVDP NQGIFAQNAQ GRHIVSPPSM
TaCCA1  QDAYRSSLNM SSTFSNMLVS TLLSNPTVHA AARLAASYWP AADSNIPVGP NQEVFAENAQ GRHIGSPPSM
BdCCA1  QDAYKSSVNM SSAFSSMLVS TLLSNPAVHA AARLAASYWP SADS--PVDP NQESPSDNAQ GRHIGSPPSM
OsCCA1  QDAYRSFANM SSTFSSMLVS TLLSNPAIHA AARLAASYWP TVDGNTP-DP NQENLSESAQ GSHAGSPPNM
ZmCCA1  QDTHRSLVNM SSTFSSMLVS TLLSNPAIHA AARLAASYWP AAEGNTPIDP NQENLADGVQ GRSIGSPPSM
SbCCA1  QDTYRSFVNM SSTFSSMLVS TLLSNPAIHA AARLAASYWP AAEGNTLIDP NQENPADDVQ GRNIGSPPSM
AtCCA1  AGDYQS---- ---FPNHIMS TLLQTPALYT AATFASSFWP PD-------- ----SSGGSP VPGNS-PPNL
AtLHY   QDDYRSFLQI SSTFSNLIMS TLLQNPAAHA AATFAASVWP YASV------ ---GNSGDSS TPMSSSPPSI

               430        440        450        460        470        480        490
        ....|....| ....|....| ....|....| ....|....| ....|....| ....|....| ....|....|
HvCCA1  ASVVAATVAA ASAWWATQGL LPLFAPPMAF PFVPVPTASF -PTADVQRAT EN---CPVDN APKECQVAQG
TaCCA1  ASVVAATVAA ASAWWATQGL LPLFAPPMAF PFVPVPTASF -PTTDVQRAT EN---WPVDN APKECQVAQE
BdCCA1  ASIVAATVAA ASAWWATQGL LPLFAPPMAF PFVPVPSASF -PTADVQRAS EN---FPVDC TPKECQVAQK
OsCCA1  ASIVTATVAA ASAWWATQGL LPLFPPPIAF PFVPAPSAPF -STADVQRAQ EKDIDCPMDN AQKELQETRK
ZmCCA1  ASIVAATVAA ASAWWATQGL LPFFAPPMAF SFVPAPSAAF -PTVDVPRPS EKDRDCPAEN AQKECQEARK
SbCCA1  ASIVAATVAA ASAWWATQGL LPFFTPPMAF PFVPAPSAAF -PTVDVPRPS EKDRDFPVEN AQKECQEAQK
```

```
AtCCA1  AAMAAATVAA ASAWWAANGL LPLCAPLSSG GFTSHPPSTF GPSCDVEYTK AS-------T LQHGSVQSRE
AtLHY   TAIAAATVAA ATAWWASHGL LPVCAPAPIT CVPFSTVAVP TPAMTEMDTV EN-------T QPFEKQNTAL

                  500       510       520       530       540       550       560
        ....|....| ....|....| ....|....| ....|....| ....|....| ....|....| ....|....|
HvCCA1  QGQPEAMIVV ASSGSGESGK GEVCPHTELN ISLADKAETT PATGAETSDA LGNKKKQDRS SCGSNTPSSS
TaCCA1  QGQPEAMIVG ASSGSDESGK GEVSPHT--- ----DKVETT PPTGAETSDA FGNKKKQDRS SCGSNTPSSS
BdCCA1  QGQPEAVMVV ASSQSDESGN GEVSLHTEIK ISPTDKAEIT PATGADTSDA FGNKKKQDRS SCGSNTPSSS
OsCCA1  QDNFEAMKVI VSSETDESGK GEVSLHTELK ISPADKADTK PAAGAETSDV FGNKKKQDRS SCGSNTPSSS
ZmCCA1  QGQLEGFRVT ASSEYDGSGK GEVSVHTELK ISPVQNADAT SAAGADTTDA FMNKKKQDRS SCGSNTPSSS
SbCCA1  QGQFEGLRVA ASSVSDGSGK GEVSLHTELK ISPVQNADAT PTTGADTSDA FRNKKKQDRS SCGSNTPSSS
AtCCA1  QEHSEASK-- ARSSLDSED- ----VENKSK PVCHEQPSAT PESDAKGSDG AGDRKQVDRS SCGSNTPSSS
AtLHY   QDQNLASKSP ASSSDDSDET GVTKLNADSK TNDDKIEEVV VTAAVHDSNT AQKKNLVDRS SCGSNTPSGS

                  570       580       590       600       610       620       630
        ....|....| ....|....| ....|....| ....|....| ....|....| ....|....| ....|....|
HvCCA1  DVEAEHVPEN QDQAND---K TQQACCSNSS AGDMNHRRFR NISS------ TNDSWKEVSE EGRMAFDKLF
TaCCA1  DVEAEHVPEN QDQAND---K TQQACCSNSS AGDMNHRRFR NISS------ TNDSWKEVSE EGRMAFDKLF
BdCCA1  DVEAEHVPEK QDQVND---K EQQASCNNLS AGDTNHRRFR SIAS------ TSESWKEVSE EGRMAFDALF
OsCCA1  DIEADNAPEN QEKAND---K AKQASCSNSS AGDNNHRRFR SSAS------ TSDSWKEVSE EGRLAFDALF
ZmCCA1  DVDVDNVPEK EGNANE---K AKQASCSNSS AGDTNHRRFR SSGS------ TSDSWKEVSE EGRLVFHALF
SbCCA1  DVDADNVPEK EDNANE---K VKQASCSNSS AGDTNHRRFR SSGS------ TSDSWKEVSE EGRLAFDALF
AtCCA1  DDVEADASER QEDGTNGEVK ETNEDTNKPQ TSESNARRSR ISSN------ ITDPWKSVSD EGRIAFQALF
AtLHY   D-AETDALDK MEKDKE-DVK ETDE--NQPD VIELNNRKIK MRDNNSNNNA TTDSWKEVSE EGRIAFQALF

                  640       650       660       670       680       690       700
        ....|....| ....|....| ....|....| ....|....| ....|....| ....|....| ....|....|
HvCCA1  SRGKLPQSFS PPQAEG---- LKVVPRGEQD EATTVTVDLN KSAAVMXHEL DTLVGPRASF PIELSHXNMK
TaCCA1  SRGKLPQSFS PPQAEG---- LKVVPRGEQD EATTVTVDLN KSAAVMDHEL DTLVGPRATF PIELSHLNMK
BdCCA1  SRGKLPQSFS PPQAEGSKAV SKEVSKEDQG EAITVTVDLN ECASIIDREL DTSVGPIASL PIELSHLNMK
OsCCA1  SRERLPQSFS PPQVEG---- SKEISKEEED EVTTVTVDLN KNAAIIDQEL DTADEPRASF PNELSNLKLK
ZmCCA1  SREKLPQSFS PPQAEG---- SKEVGKKEED EVTTVAVDLN KSTTSIDHDL DTIGEPRASF PNELSPLKLK
SbCCA1  SREKLPQSFS PPQAED---- SKEVAKEEEN EVTTVAVDLN KNATSIDHDL DTMDEPRASF PNELSHLKLK
AtCCA1  SREVLPQSFT YREEHR---- --EEEQQQQE QRYPMALDLN FTAQLTPVDD QEEKRNTGFL GIGLDASKLM
AtLHY   ARERLPQSFS PPQVAE---- --NVNRKQSD TSMPLAP--N FKSQDSCAAD QE-----GVV MIGVGTCKSL

                  710       720       730       740
        ....|....| ....|....| ....|....| ....|....| ....|..
HvCCA1  SR-RTGFKPY KRCSVEAKEN RVPAAD---- --EVGTKRIR LDSEPST
TaCCA1  SR-RTGFKPY KRCSVEAKEN RVPAAD---- --EVGTKRIR LDSEPST
BdCCA1  SR-RTGFKPY KRCSVEAKEN RVPASD---- --EVGTKRIR LDSEAST
OsCCA1  SR-RTGFKPY KRCSVEAKEN RVPASD---- --EVGTKRIR LESEAST
ZmCCA1  SR-QTGFKPY KRCSVEAKEN RVPASD---- --EVGTKRIR LDSEAST
SbCCA1  SR-RTGFKPY KRCSVEAKEN RVPASD---- --EVGTKRIR LESEAST
AtCCA1  SRGRTGFKPY KRCSMEAKES RILNNNPIIH VEQKDPKRMR LETQAST
AtLHY   KTRQTGFKPY KRCSMEVKES QVGNIN--NQ SDEKVCKRLR LEGEAST
```
