# Supplementary material for: Expression conservation within the circadian clock of a monocot: natural variation at barley Ppd-H1 affects circadian expression of flowering time genes, but not clock orthologs
Source: BMC Plant Biol. 2012 Jun 21;12:97. doi: 10.1186/1471-2229-12-97 (PMC3478166; doi:10.1186/1471-2229-12-97)
Supplement: Additional file 4 — Protein alignment of PRR gene family. Asterisks and circles indicate the conserved Pseudo receiver and CCT domains, respectively. [file 1471-2229-12-97-S4.pdf]

```
                 10         20         30         40         50         60         70
         ....|....| ....|....| ....|....| ....|....| ....|....| ....|....| ....|....|
HvPRR1   ---------- ---------- ---------- ---------- ---------- ---------- ----------
TaPRR1   ---------- ---------- ---------- ---------- ---------- ---------- ----------
BdPRR1   ---------- ---------- ---------- ---------- ---------- ---------- ----------
OsPRR1   ---------- ---------- ---------- ---------- ---------- ---------- ----------
ZmPRR1   ---------- ---------- ---------- ---------- ---------- ---------- ----------
SbPRR1   ---------- ---------- ---------- ---------- ---------- ---------- ----------
AtPRR1   ---------- ---------- ---------- ---------- ---------- ---------- ----------
HvPRR37  ---------- ---------- ---------- ---------- ---------- ---------- ----------
TaPRR37  ---------- ---------- ---------- ---------- ---------- ---------- ----------
BdPRR37  ---------- ---------- ---------- ---------- ---------- ---------- ----------
OsPRR37  ---------- ---------- ---------- ---------- ---------- ---------- ----------
ZmPRR37  ---------- ---------- ---------- ---------- ---------- ---------- ----------
SbPRR37  ---------- ---------- ---------- ---------- ---------- ---------- ----------
HvPRR73  ---------- ---------- ---------- ---------- ---------- ---------- ----------
TaPRR73  ---------- ---------- ---------- ---------- ---------- ---------- ----------
BdPRR73  ---------- ---------- ---------- ---------- ---------- ---------- ----------
OsPRR73  ---------- ---------- ---------- ---------- ---------- ---------- ----------
ZmPRR73  ---------- ---------- ---------- ---------- ---------- ---------- ----------
SbPRR73  ---------- ---------- ---------- ---------- ---------- ---------- ----------
AtPRR3   ---------- ---------- ---------- ---------- ---------- ---------- ----------
AtPRR7   ---------- ---------- ---------- ---------- ---------- ---------- ----------
HvPRR59  ---------- ---------- ---------- ---------- ---------- ---------- ----------
TaPRR59  ---------- ---------- ---------- ---------- ---------- ---------- ----------
BdPRR59  ---------- ---------- ---------- ---------- ---------- ---------- ----------
OsPRR59  ---------- ---------- ---------- ---------- ---------- ---------- ----------
ZmPRR59  ---------- ---------- ---------- ---------- ---------- ---------- ----------
SbPRR59  ---------- ---------- ---------- ---------- ---------- ---------- ----------
HvPRR95  ---------- ---------- ---------- ---------- ---------- ---------- ----------
TaPRR95  ---------- ---------- ---------- ---------- ---------- ---------- ----------
BdPRR95  ---------- ---------- ---------- ---------- ---------- ---------- ----------
OsPRR95  ---------- ---------- ---------- ---------- ---------- ---------- ----------
ZmPRR95  ---------- ---------- ---------- ---------- ---------- ---------- ----------
SbPRR95  ---------- ---------- ---------- ---------- ---------- ---------- ----------
AtPRR5   MWQTWPRQPI LLDIFSNPNT LSTTVRSWSV RHPLSIITVK TFARFFLDIF FSPHYYRKNK VLFFALFSFI
AtPRR9   ---------- ---------- ---------- ---------- ---------- ---------- ----------

                 80         90        100        110        120        130        140
         ....|....| ....|....| ....|....| ....|....| ....|....| ....|....| ....|....|
HvPRR1   ---------- ---------- ---------- ---------- --------MV GAGEGGRAGG SGAGVG----
TaPRR1   ---------- ---------- ---------- ---------- --------MV GAGEGVRAGG SGAGVG----
BdPRR1   ---------- ---------- ---------- ---------- --------MV GAGEGGRVGG SGSGSG----
OsPRR1   ---------- ---------- ---------- ---------- --------MV GAGEGDRVGG G---------
ZmPRR1   ---------- ---------- ---------- ---------- --------MA GGGEGDRVG- ----------
SbPRR1   ---------- ---------- ---------- ---------- --------MV GGGEGDRVGG GGGGGGG---
AtPRR1   ---------- ---------- ---------- ---------- --------MD LNGE------ ----------
HvPRR37  ---------- ---------- MFPLGAR--- ---------- ----QPPPSA MDNHQQQQPP --RGE-----
TaPRR37  ---------- ---------- ---------- ---------- ---------- MDRHHHQQPP SPQGE-----
BdPRR37  ---------- ---------- ---------- ---------- ---------- MD--DAAQPP ----------
OsPRR37  -----MMGTA HHN--QTAGS ALGVGVG--- ---------- ----DANDAV PGAGGGGYSD PDGGPIS---
ZmPRR37  -----MMGTA HHN--QTAGS ALGVGVG--- ---------- ----DANDAV PGAGGGGYSD PDGGPIS---
SbPRR37  -----MMLRN NNNNLRSNGP SDGLLSR--- ---------- ----PTPAVL QDDDDGGDDD TEN-------
HvPRR73  MVSAGQAGAD GPSSSDIRGI GNGAVENQNG HALKPNEDKE WRGGSKEEDW PSTHSAPPG- --LDEHK---
TaPRR73  MVSAGQAGAD GPSTSDIRGT GNGAVEN--G HALKANEDKE WRGGSKEEDW PSTHSAPPG- --LDEHK---
BdPRR73  MVGACQAATD GPSSYAVRGI GNSATEN--G HALKAKEEKE WRSGN-DEDL PNGHSAPPGA QQINEQK---
OsPRR73  MGSACEAGTD EPSRDDVKGT GNGILEN--G HSHK-PEEEE WRNGMGEDLP N-GHSTPP-E PQQTDEQ---
ZmPRR73  MGSACQAGTD GPSRKDVLGI GNAALEN--G HHQAEADADE WRE-KEEDLA NNGHSAPPPG MQQVDEH---
SbPRR73  MGSACQAGMD GPSRKDVLGI GNVALEN--G HHEVGADADE WRE-KEEDLA N-GHSAPP-G MQQVDEQ---
AtPRR3   -MCFNNIETG DEVETERQVF GSSEED---- --------EF RVEDTARN-- TNNVQISQQQ ----------
AtPRR7   -MNANEEGEG SRYPITDRKT GETKFDR--- -------VES RTEKHSEEEK TNGITMDVRN GSSGGLQIPL
HvPRR59  ---------- ---------- ---------- --MSPDADAG EPAAAAAAA- ---------- --------GG
TaPRR59  ---------- ---------- ---------- ---------- ---------- ---------- ----------
BdPRR59  ---------- ---------- ---------- --MSPDADGG EAAAAAAAVE KGSGGGGGEV EGGGGGVAAG
OsPRR59  ---------- ---------- ---------- --MSPDADAA AAAAAGGEGA AAAG------ -------VGT
ZmPRR59  ---------- ---------- ---------- --MSPDADAA AGGGGG--GE ASAGAGAG-- ASSPPASASA
SbPRR59  ---------- ---------- ---------- --MSPDADAT AGAGGGDGGG GEAGGGAG-- AGASSSPASA
HvPRR95  ---------- ---------- ---------- --------MA RRGQGQGGG- VEEREVV--- DQDGRE----
TaPRR95  ---------- ---------- ---------- --------MA RGGQGQGGGG VEEREVVNVV DQDGRE----
BdPRR95  ---------- ---------- ---------- ---------- ---MGRGGGG VEDREVVNVE DQGETG----
OsPRR95  ---------- ---------- ---------- ---------- ---MG-GGVE --ERKVVDLE DGDGEEGEDA
ZmPRR95  ---------- ---------- ---------- ---------- ---MG-GGLD EAVK-VVDLE DGEGEE-EAE
SbPRR95  ---------- ---------- ---------- ---------- ---MG-GGVD EVVKVVVDLE DGEGEE-DAE
AtPRR5   SPLTNILICF VTVSLSLELS SSSSIIDLGF SKLSVCVVIM TSSEEVVEVT VVKAPEAGGG KLSRRKIRKK
AtPRR9   ---------- ---------- ---------- ---------- -----MGEIV VLSSDDG--- -METIKNRVK
```

```
                 150        160        170        180        190        200        210
          ....|....| ....|....| ....|....| ....|....| ....|....| ....|....| ....|....|
                                  ****************************************************
HvPRR1    ----AGAG-- QPFVDRSKVR ILLCDSDPDS SQDVLRLLCN CSYQVTCAKS PRQVINVLNC EGAEIDIILA
TaPRR1    ----AGAGAG QPFVDRSKVR ILLCDSDPDS SQDVLRLLCN CSYQVTCAKS PRQVINVLNC EGAEIDIILA
BdPRR1    ----AGVGGG QSFVDRSKVR ILLCDSDPDS SRDVLRLLRN CSYQVTCAKS PRQVINVLNC EGAEMDIILA
OsPRR1    ----AAVGGG QQFVDRSKVR ILLCDSDPSS SREVLRLLCN CSYQVTCAKS PRQVINVLNC EAGEIDIILA
ZmPRR1    ------VGGA QQFVDRSKVR ILLCDGDATS SREVLRLLCN CAYHVTCAKS PRQVVNILNY EGGEIDIILA
SbPRR1    ----LGVGGG QQFVDRSKVR ILLCDGDATS SREVLRLLCN CSYHVTCAKS PRQVINILNY EGGEIDIILA
AtPRR1    ------CKGG DGFIDRSRVR ILLCDNDSTS LGEVFTLLSE CSYQVTAVKS ARQVIDALNA EGPDIDIILA
HvPRR37   --HAAQPRCW EEFLHRKTIR VLLVETDDST RQVVAALLRH CMYQVIPVEN GHQAWAYLQD MQSNIDLVLT
TaPRR37   --HAAQPRCW EEFLHRKTIR VLLVETDDST RQVVTALLRH CMYQVIPAEN GHQAWAYLQD MQSNIDLVLT
BdPRR37   ------LCTW EHYIHKKIIR VLLVETDDST RQIVTALLRH CMYQVIPAEN GDQAWARLQD MQDNIDLVLS
OsPRR37   GVQRPPQVCW ERFIQKKTIK VLLVDSDDST RQVVSALLRH CMYEVIPAEN GQQAWTYLED MQNSIDLVLT
ZmPRR37   GVQRPPQVCW ERFIQKKTIK VLLVDSDDST RQVVSALLRH CMYEVIPAEN GQQAWTYLED MQNSIDLVLT
SbPRR37   --QQQEAVYW ERFLQKKTIN VLLVESDDCT RRVVSALLRH CMYQVN---- ---------- ----------
HvPRR73   QQQQDRVIRW EKFLPVKTLR VLLVENDDCT RHVVRALLRK CGYEVISAEN GLDAWQYLED VQNRIDLVLT
TaPRR73   QQQ-GRVIRW EKFLPVETLR VLLVENDDCT RHVVRALLRK CGYEVIAAEN GLHAWHYLED VQNRIDLVLT
BdPRR73   EQQ-GRVIRW ERFLPVKTLR VLLVENDDCT RQVVGALLRK CGYEVISAEN GLHAWQYLED LQNRIDLVLT
OsPRR73   KEHQVQIVRW ERFLPVKTLR VLLVENDDST RQVVSALLRK CCYEVIPAEN GLHAWQCLED LQNHIDLVLT
ZmPRR73   KEEQRQSIHW ERFLPVKTLR VLLVENDDST RQVVSALLRK CCYEVIPAEN GLHAWRYLED LQNNIDLVLT
SbPRR73   -EQQGQSIHW ERFLPVKTLR VMLVENDDST RQVVSALLRK CCYEVIPAEN GSHAWRYLED LQNNIDLVLT
AtPRR3    QQPLAHVVKW ERYLPVRSLK VLLVENDDST RHIVTALLKN CSYEVTAVPD VLEAWRILED EKSCIDLVLT
AtPRR7    SQQTAATVCW ERFLHVRTIR VLLVENDDCT RYIVTALLRN CSYEVVEASN GIQAWKVLED LNNHIDIVLT
HvPRR59   GGAARGVIRW DEILPRRSLR VLLVEHDDST RQVVTALLRK CGYRVAAVAD GMKAWEVMRG RAYAFDLVLT
TaPRR59   ---------- ---------- ---------- ---------- ---------- ---------- ----------
BdPRR59   GGAARGVIRW DEILPRRSLR VLLVEHDDST RQVVTALLRK CGYRVAAVAD GMKAWEVMRG RAYAFDLVLT
OsPRR59   AGEGRGVIRW DQILPRRSLR VLLVEHDDST RQVVTALLRK CGYRVAAVAD GMKAWGVMRE RAYAFDLVLT
ZmPRR59   AANGRALVRW DQILPRRSLR VLLVEHDDST RQIVTALLRK CGYRVAAVAD GMKAWEVMRE RAYDFDLVLT
SbPRR59   AANGRALVRW DQILPRRSLR VLLVEHDDST RQVVTALLRK CGYRVAAVAD GMKAWEVMRG RAYAFDLVLT
HvPRR95   -------EEV RRALPMMPVR VLLAEGDDST RHVISALLRK CGYHVSAASD GVKAWELLKE KSFKIDLVLT
TaPRR95   -------EEV RRALPMMPVR VLLAEGDDST RHVISALLRK CGYHVSAASD GVKAWELLKE KSFKIDLVLT
BdPRR95   -------QEA LRALPMMPVR VLLAEGDDST RHVISALLRK CGYHVAAASD GVKAWEILKE KSFNIDLVLT
OsPRR95   AAVAAGSSRE TRMLPRMPVR VLLAEGDDST RHIICALLRK CGYRVAAASD GVKAWDILKE KSFNIDLVLT
ZmPRR95   AAAAEGSSME MGMLPRMPVR VLLAEGDDST RHVISALLRK CGYRVAAASD GVKAWDLLKE KSFNVDLVLT
SbPRR95   AAAAEGSSRE TRMLPRMPVR VLLAEGDDST RHVISALLRK CGYRVAAASD GVKAWDILKE KSFNIDLVLT
AtPRR5    DAGVDGLVKW ERFLPKIALR VLLVEADDST RQIIAALLRK CSYRVAAVPD GLKAWEMLKG KPESVDLILT
AtPRR9    SS---EVVQW EKYLPKTVLR VLLVESDYST RQIITALLRK CCYKVVAVSD GLAAWEVLKE KSHNIDLILT

                 220        230        240        250        260        270        280
          ....|....| ....|....| ....|....| ....|....| ....|....| ....|....| ....|....|
          ****************************************************************************
HvPRR1    EVDLPV-SKC FKMLKYIGRN KELRHIPIIM MSNRDEVSVV VKCLRLGAAE YLVKPLRMNE LLNLWTHVWR
TaPRR1    EVDLPV-SKC FKMLKYIGRN KELRHIPIIM MSNRDEVSVV VKCLRLGAAE YLVKPLRMNE LLNLWTHVWR
BdPRR1    EVDLPV-SKC FKMLKYIARN KELRHIPIIM MSNRDEVSVV VKCLRLGAAE YLVKPLRMNE LLNLWTHVWR
OsPRR1    EVDLPV-SKC FKMLKYIARN KELRHIPIIM MSNRDEVSVV VKCLRLGAAE YLVKPLRMNE LLNLWTHVWR
ZmPRR1    EVDLPV-SKC FKMLKYIARN KDLRHIPIIM MSNRDEVSVV VKCLRLGAAE YLVKPLRTNE LLNLWTHVWR
SbPRR1    EVDLPV-SKC FKMLKYIARN KDLRHIPIIM MSNRDEVSVV VKCLRLGAAE YLVKPLRTNE LLNLWTHVWR
AtPRR1    EIDLPM-AKG MKMLRYITRD KDLRRIPVIM MSRQDEVPVV VKCLKLGAAD YLVKPLRTNE LLNLWTHMWR
HvPRR37   EVFMHGGLSG IDLLGRIMNH EVCKDIPVIM MSSHDSMGTV LSCLSNGAAD FLAKPIRKNE LKNLWAHVWR
TaPRR37   EVFMHGGLSG IDLLGRIMNH EVCKDIPVIM MSSHDSMGTV LSCLSNGAAD FLAKPIRKNE LKNLWAHVWR
BdPRR37   EVVMPG-LSG ISLLEKIMAH NVCKDIPVIM MSSNDAMGTV FKCLSTGAVD FLVKPIRKNE LKNLWQHVWR
OsPRR37   EVVMPG-VSG ISLLSRIMNH NICKNIPVIM MSSNDAMGTV FKCLSKGAVD FLVKPIRKNE LKNLWQHVWR
ZmPRR37   EVVMPG-VSG ISLLSRIMNH NICKNIPVIM MSSNDAMGTV FKCLSKGAVD FLVKPIRKNE LKNLWQHVWR
SbPRR37   ---------- ---------- ---------- ---------- ---------- ----PIRKNE LKNLWQH---
HvPRR73   EVAMPC-LSG IGLLSKITSH SICKGIPVIM MSKNDSMSTV FKCLSKGAVD FLVKPIRKNE LKTLWQHIWR
TaPRR73   EVAMPC-LSG IGLLSKITSH SICKGIPVIM MSKNDSMSTV FRCLSKGAVD FLVKPIRKNE LKTLWQHIWR
BdPRR73   EVAMPC-LSG ISLLSKIMSH KICKDIPVIM MSKNDSMGTV FKCLSKGAVD FLVKPIRKNE LKNLWQHIWR
OsPRR73   EVVMPR-LSG IGLLSKITSH KICKDIPVIM MSSNDSMGTV FKCLSKGAVD FLVKPIRKNE LKNLWQHVWR
ZmPRR73   EVFMPC-LSG IGLLSKITSH KICKDIPVIM MSTNDSMSMV FKCLSKGAVD FLVKPLRKNE LKNLWQHVWR
SbPRR73   EVFMPC-LSG IGLLSKITSH KICKDIPVIM MSSNDSMSMV FKCLSKGAVD FLVKPLRKNE LKNLWQHVWR
AtPRR3    EVDMPV-HSG TGLLSKIMSH KTLKNIPVIM MSSHDSMVLV FKCLSNGAVD FLVKPIRKNE LKNLWQHVWR
AtPRR7    EVIMPY-LSG IGLLCKILNH KSRRNIPVIM MSSHDSMGLV FKCLSKGAVD FLVKPIRKNE LKILWQHVWR
HvPRR59   EVNMPT-LSG IDLLSRIVAA DECKNIPVIM MSSQDSIGTV LKCMQNGAVD FLVKPVRKNE LRNLWQHVWR
TaPRR59   ---------- ---------- --------MM MSSQDSIGTV LKCMQNGAVD FLVKPVRKNE LRNLWQHVWR
BdPRR59   EVDMPT-LSG IDLLARIVAA HECKNIPVIM MSSQDSIGTV LRCMQNGAVD FLVKPVRKNE LRNLWQHVWR
OsPRR59   EVTMPT-LSG IELLSRIVAS DECKNIPVIM MSSQDSIGTV LRCMQKGAVD FLVKPVRKNE LRNLWQHVWR
ZmPRR59   EVAMPS-LSG IQLLSRIAAA AECKNIPVIM MSSQDSIGTV LKCMQKGAVD FLVKPVRKNE LGNLWQHVWR
SbPRR59   EVAMPS-LSG IQLLSRIVAA DECKNIPVIM MSSQDSIGTV LKCMQKGAVD FLVKPVRKNE LRNLWQHVWR
HvPRR95   EVELPS-MSG FLLLSTIMEH DACKNIPVIM MSSNDAVSMV FKCMLKGAAD FLVKPIRKNE LRNLWQHVWR
TaPRR95   EVELPL-MSG FLLLSTIMEH DACKNIPVIM MSSNDAVSMV FKCMLKGAAD FLVKPIRKNE LRNLWQHVWR
BdPRR95   EVELPA-MSG FLLLSTIMEH EACKNIPVIM MSSNDAVSMV FKCMLKGAAD FLVKPIRKNE LRNLWQHVWR
OsPRR95   EVELPL-MSG FLLLSTIMEH DACKNIPVIM MSSNDSVSMV FKCMLKGAAD FLVKPIRKNE LRNLWQHVWR
ZmPRR95   EVELPL-MSG FLLLSTIMEH DASKNIPVIM MSSHDSVSMV FKCMLKGAAD FLVKPIRKNE LRNLWQHVWR
SbPRR95   EVDLPL-MSG FLLLSTIMEH DASKNIPVIM MSSHDSVSMV FKCMLKGAAD FLVKPIRKNE LRNLWQHVWR
AtPRR5    EVDLPS-ISG YALLTLIMEH DICKNIPVIM MSTQDSVNTV YKCMLKGAAD YLVKPLRRNE LRNLWQHVWR
AtPRR9    ELDLPS-ISG FALLALVMEH EACKNIPVIM MSSQDSIKMV LKCMLRGAAD YLIKPMRKNE LKNLWQHVWR
```

```
                 290       300       310       320       330       340       350
         ....|....| ....|....| ....|....| ....|....| ....|....| ....|....| ....|....|
HvPRR1   RRRMLG---- ---------- ---------- -------LAE KNFFIDNLEL VLSEPSDANT NSTT------
TaPRR1   RRRMLG---- ---------- ---------- -------LAE KNFFIDNLEL VLSEPSDANT NSTT------
BdPRR1   RRRMLG---- ---------- ---------- -------LAE KNFFIDNLEL VLSDPSDANT NSTT------
OsPRR1   RRRMLG---- ---------- ---------- -------LSE KNFFNDNFEL ALSEPSDANT NSTT------
ZmPRR1   RRRMLG---- ---------- ---------- -------LPE KNFFNDNFEL VLSEPSDANT NSTT------
SbPRR1   RRRMLG---- ---------- ---------- -------LPE KNFFHDNFEL VLSEPSDANT NSTT------
AtPRR1   RRRMLG---- ---------- ---------- -------LAE KNMLSYDFDL VGSDQSDPNT NSTN------
HvPRR37  RSHS------ ---------- ---------- -SSGSGSGS- ---AIQTQKC TKSKSGDDSN NNS-------
TaPRR37  RSHS------ ---------- ---------- -SSGSGSGS- ---AIQTQKC TKSKSGDDSN NNS-------
BdPRR37  RCHS------ ---------- ---------- -SSGSGSGSG SESGIQTQKC TKSKSANESD NNSG------
OsPRR37  RCHS------ ---------- ---------- -SSGSGSES- ---GIQTQKC AKSKSGDESN NNNG------
ZmPRR37  RCHS------ ---------- ---------- -SSGSGSES- ---GIQTQKC AKSKSGDESN NNSG------
SbPRR37  ---------- ---------- ---------- -SSGSGSES- ---GIQTQKC GKSKGGKESG NNSG------
HvPRR73  RCHS------ ---------- ---------- -SSGS--ES- ---GIHIQKC SKPKTGDEYA KNSG------
TaPRR73  RCHS------ ---------- ---------- -SSGS--ES- ---GIHTQKC SKPKAGDEYE NNSHDDDDDC
BdPRR73  RCHS------ ---------- ---------- -SSGS--ES- ---GIHTQKC SKPKTGDEYE NNSG------
OsPRR73  RCHS------ ---------- ---------- -SSGSGSES- ---GIRTQKC TKPKVDDEYE NNSG------
ZmPRR73  RCHS------ ---------- ---------- -SSGS--ES- ---GIQTQKC AKLNTGDEYE NGSD------
SbPRR73  RCHS------ ---------- ---------- -SSGSGSES- ---GIQTQKC AKPNTGDEYE NDSD------
AtPRR3   RCHSVRVLIW LPVLQKLLCV FFLLMRLLIY QSSGSGSESG ---IHD-KKS VKPESTQGSE NDAS------
AtPRR7   RCQS------ ---------- ---------- -SSGSGSESG ---THQTQKS VKSKSIKKSD QDSG------
HvPRR59  RHSM------ ---------- ---------- ---------- ---NSQLNAS ENNAASNHIS VNSG------
TaPRR59  RHSM------ ---------- ---------- ---------- ---NSQLNAS ENNAASNHIS VNSG------
BdPRR59  RHSM------ ---------- ---------- ---------- ---NTQTNAS ENNAASNHIS ANSG------
OsPRR59  RHAM------ ---------- ---------- ---------- ---NSQTNAS ENNAASNHLS ANGG------
ZmPRR59  RHAM------ ---------- ---------- ---------- ---NCQTNGS ENNAASNHVS TNVA------
SbPRR59  RHAM------ ---------- ---------- ---------- ---NCQTNGS ENNAASNHIS ANVA------
HvPRR95  KQLA------ ---------- ---------- ---------- ---NGEIDVQ QIQQEENVAE QHGR------
TaPRR95  KQLA------ ---------- ---------- ---------- ---NGEIDVQ QIQQEENAPE QHGQ------
BdPRR95  KQLS------ ---------- ---------- ---------- ---NGGL-VQ HTQQEDKLTE WQGQ------
OsPRR95  KQLS------ ---------- ---------- ---------- ---SGVLDVQ HTQQEDNLTE RHEQ------
ZmPRR95  KQLA------ ---------- ---------- ---------- ---NGGPNVQ HIQREENLAE RIQQ------
SbPRR95  KQLA------ ---------- ---------- ---------- ---NGGSDVH HIQQEENLAE RIEQ------
AtPRR5   RQTS------ ---------- ---------- ---------- ---------- -LAPDSFPWN ESVG------
AtPRR9   RLT------- ---------- ---------- ---------- ---------- -LRDDPTAHA QSLP------

                 360       370       380       390       400       410       420
         ....|....| ....|....| ....|....| ....|....| ....|....| ....|....| ....|....|
HvPRR1   ---------- ---------- --LLSDETDD KPKGNRNHET NTSSQYEYES --PATDPPKT D---------
TaPRR1   ---------- ---------- --LLSDETDD KPKGNRNHET NTSSQHEYES --PAVDPPKT D---------
BdPRR1   ---------- ---------- --LLSDETDD RPKENTNHET NTSNQHEYES --PVAEFPKR D---------
OsPRR1   ---------- ---------- --LLSDDTDD KPKENINQET STSNQHEYES NPSDAEPKQK G---------
ZmPRR1   ---------- ---------- --LLSDETDD RPKGNTNQET GTSKQLEYES NPSVAEPDQR E---------
SbPRR1   ---------- ---------- --LLSDETDD RPKENMNQET GTSNQLEYES NPSVAEPDQR D---------
AtPRR1   ---------- ---------- --LFSDDTDD RSLRSTNPQR GNLSHQENEW SVATAPVHAR DG--------
HvPRR37  ----NDRNDD -------ASM GLNARD-GSD NGSGTQAQSS WTKRAVEIDS PQDMSPDQSA DP-PEGTCAH
TaPRR37  ----NNRNDD -------ASM GLNARD-GSD NGSGT--QSS WTKRAVEIDS PQDMSPDQSV DP-PDSTCAH
BdPRR37  ---SNDRNDD -------ISM GLNARD-GSD NGSGT--QSS WTKLGVEIDS PQDMSPDHSA DP-PDSTCAH
OsPRR37  ---SNDDDDD DG-----VIM GLNARD-GSD NGSGTQAQSS WTKRAVEIDS PQAMSPDQLA DP-PDSTCAQ
ZmPRR37  ---SNDDDDD DG-----VIM GLNARD-GSD NGSGTQAQSS WTKRAVEIDS PQAMSPDQLA DP-PDSTCAQ
SbPRR37  ---SNDSHDN -E-----ADM GLNARD-DSD NGSGTQAQSS WTKCAVEMDS PQAMSLDQLA DS-PDSTY--
HvPRR73  GSHDDDDDDD ---ADDDFSV GPNARD-GSD NGSGT--QSS WTKRAVEIDS PQLVSSDHLS DS-PDSTCAQ
TaPRR73  GSHDDDDDDD DD-ADDDFSV GPNARD-GSD NGSGT--QSS WTKRAVEIDS PQLLSSDHLA ES-PDSTCAQ
BdPRR73  SSHDDDDGDD ---SDDDFNV GLSARD-SSD NGSGT--QSS WTKRAVEIDS PQSMSPDQLA DSSPDSTCAQ
OsPRR73  SNNDNEDDDD NDEDDDDLSV GHNARD-GSD NGSGT--QSS WTKRAVEIDS PQQMSPDQPS DL-PDSTCAQ
ZmPRR73  SNHDDEENDD GD--DDDFSV GLNARD-GSD NGSGT--QSS WTKRAVEIDS PQPISPDQLV DP-PDSTCAQ
SbPRR73  SNHDDEENDE DD--DDDFSV GLNARD-GSD NGSGT--QSS WTKRAVEIDS PEPMSPDQLA DP-PDSTCAQ
AtPRR3   --ISDEHRNE SG-----SSG GLSNQDGGSD NGSGT--QSS WTKRASDTKS TS-------- ----------
AtPRR7   --SSDE--NE NG-----SIG -LNASDGSSD -GSGA--QSS WTKKAVDVDD SPRAVS--LW DR-VDSTCAQ
HvPRR59  ---------- ---------- --TGSKTGEN SDEESDAQSS GSKRETEIQS VEKLPETVTH N---------
TaPRR59  ---------- ---------- --SGSKTGEN SDEESDAQSS GSKRETEIQI VEKLPEIVTH N---------
BdPRR59  ---------- ---------- --NRSKTGDN SDEESDAQSS GSKRETEIQS VEKLPETVTE N---------
OsPRR59  ---------- ---------- --NGSKTGEH SDEESDAQSS GSKREVEIQS AEKLPEVVAD G---------
ZmPRR59  ---------- ---------- --NGSKTGEN NDEESDAQSF GNKRETEIKS AETLPDIRRD E---------
SbPRR59  ---------- ---------- --NGSKTGEN SDEESDAQSF GSKRETEIQS VEKLPDIRRD ED--------
HvPRR95  ---------- ---------- --KTEATKAE HSTQNVVRKN RECSEQESDA QSSCTRSEPE A---------
TaPRR95  ---------- ---------- --KTEVTKAE HSTQNVVRKN RECSEQESDA QSSCTRSEPE A---------
BdPRR95  ---------- ---------- --KTGVTKAE HLIENVAHKR KECSEQESDA QSSCTRSEVE A---------
OsPRR95  ---------- ---------- --KTGVTKAE HVTENVVHKN MECSEQESDA QSSCTRSELE A---------
ZmPRR95  ---------- ---------- --KTGVTKSD NLDRDVPCKN RECSEQESDA QSSCTRSELE A---------
SbPRR95  ---------- ---------- --KTGVTKAD NLNRDGPRKN RECSEQESDA QSSCTRSELE A---------
AtPRR5   ---------- ---------- --QQKAEGAS ANNSNGKRDD HVVSGNGGDA QSSCTRPEME G---------
AtPRR9   ---------- ---------- --ASQHNLED TDETCEDSRY HSDQGSGAQA INYNGHNKLM E---------
```

```
                430        440        450        460        470        480        490
          ....|....| ....|....| ....|....| ....|....| ....|....| ....|....| ....|....|
HvPRR1    ---------- ---------- ---------- ---------- --------QL ENLPSIAEDD DNASSPGGMF
TaPRR1    ---------- ---------- ---------- ---------- --------QL ENLPSIAEDD DKASSPGGMF
BdPRR1    ---------- ---------- ---------- ---------- --------QV ENLPDIMEYD NNAPYTGGMF
OsPRR1    ---------- ---------- ---------- ---------- --------TP EGLLVSTEGG DQASSPGVMF
ZmPRR1    ---------- ---------- ---------- ---------- --------KM EGVPGSALDA SQKSSPRRAF
SbPRR1    ---------- ---------- ---------- ---------- --------KM EGVPGSVVDA SQASSPGRMF
AtPRR1    ---------- ---------- ---------G LGADGTATSS LAVTAIEPPL DHLAGSHHEP MKRNSNPAQF
HvPRR37   VNHPKSEICS NRWLPGTNNK KCQKPKETTN GDGFKGKELE IGAPGNLNTD DQSSPKESSV KPTD-GRCEY
TaPRR37   VSHLKSEICS NR-LRGTNNK KCQKPKETN- -AEFKGKELE IGAPGNLNTE DQSSPNESSV KPADNGRCEY
BdPRR37   VIHPKSEICS NRWLPGTNNK KCKKQKENN- -----GKDME KGAPSNLNAD DQSSPNERSI KPTD-GWCEY
OsPRR37   VIHLKSDICS NRWLPCTSNK NSKKQKETN- -DDFKGKDLE IGSPRNLNTA YQSSPNERSI KPTD-RRNEY
ZmPRR37   VIHLKSDICS NRWLPCTSNK NSKKQKETN- -DDFKGKDLE IGSPRNLNTA YQSSPNERSI KPTD-RRNEY
SbPRR37   ---------- ---------- ---------- --DFKEKDLE IGGPGNLYID HQSSPNERPI KATD-GRCEY
HvPRR73   VIHPRSEIGS NRWLPTANKR NINNQKEKN- -DDSMGKYLE IGAPRNSSLA YQSSPNEMSV NPTEKQHENL
TaPRR73   VIHPRSEIGS NRWLPTANKR NINNQKENN- -DDSMGKYLE IGAPRNSSLG YQSSPNEMSV NPTEKQHENL
BdPRR73   VIHPKSEIGS NRWLPTENKR NSNNQKESN- -GDSMGKYLE IGAPRNSSLG YQSSPNEVSV NPIEKQHETL
OsPRR73   VIHPTSEICS NRWLPTANKR SGKKHKENN- -DDSMGKYLE IGAPRNSSME YQSSPREMSV NPTEKQHETL
ZmPRR73   VIHPRSEICS NKWLPTANKR NVKKQKENK- -DESMGRYLG IGAPRNSSAE YQSSLNDVSV NPIEKGHENH
SbPRR73   VIHPKSEICS NKWLPTANKR NGKKHKENK- -DESMGRYLE IGAPRNSSAE YQSSLNDVSV NPTEKRHETH
AtPRR3    --------PS NQFPDAPNKK GTYENG---- ---------- ---CAHVNRL KEA-EDQ--K EQIGTGSQTG
AtPRR7    VVHSNPEFPS NQLVAPPAEK ETQEHDDKFE -DVTMGRDLE ISIRRNCDLA LEP-KDEPLS KTTGIMRQDN
HvPRR59   ---------- ---------- ---------- -----VASSP RELKVQNGPF DR-MNTKAPA LKGTDDAPSG
TaPRR59   ---------- ---------- ---------- -----GASSS RELKIQNGPF DR-INTKALA LKGTDDAPSG
BdPRR59   ---------- ---------- ---------- -----GASSS RELTIQNGPF DR-VTTKAHA FNINVDPPSG
OsPRR59   ---------- ---------- ---------- -----GAGSS REHKIQNGFI DG-MNTKSHA LKGNDDAPSG
ZmPRR59   ---------- ---------- ---------- -----MAGSS KKIELQNKSN DG-VNTKVDA SKDSDGAPSE
SbPRR59   ---------- ---------- ---------- ----EVAGSS KKTESQNKSY DVRVNTKVDA SKDSDGAPSG
HvPRR95   ---------- ---------- ---------- ----ESKHTN SFLEFKQITE RQSS-TDPKN TVEN------
TaPRR95   ---------- ---------- ---------- ----ESKHAN SFLDFKQITE RQSS-TDPKN TLEN------
BdPRR95   ---------- ---------- ---------- ----ESKHTN NFLEFKQITG KYLS-SDLKS TEDNGQTKTQ
OsPRR95   ---------- ---------- ---------- ----DSRQTN NLLEYKQPMG RHFSKPDHKN TEKNGGTKIH
ZmPRR95   ---------- ---------- ---------- ----ESKQTD NILEYKQSTQ RHLSIPSHKN VELNGQTKIQ
SbPRR95   ---------- ---------- ---------- ----ESKQTN NILEYKQSTE RHLSIPSHKN VELNGQTKIR
AtPRR5    ---------- ---------- ---------- ----ESADVE VSARDAVQM- ---------- ----------
AtPRR9    ---------- ---------- ---------- ----NGKSVD E--------- ---------- ----------

                500        510        520        530        540        550        560
          ....|....| ....|....| ....|....| ....|....| ....|....| ....|....| ....|....|
HvPRR1    S--------- --------RP IKTNLRIAES SAFLAYVKSS TPANSSLDTE LQRGANKLDI VDHQVNFSGS
TaPRR1    S--------- --------RP IKTNLRIAES SAFLAYVKSS TPANNPLDNE LQREGNQIDV MDHQGNFSGA
BdPRR1    S--------- --------RP IKTNLRVGES SAFLAYVKSS TPTDSQLDSE QPRGCDRLDS MGHQGNLSRA
OsPRR1    S--------- --------RP IKTNLRVAES SAFLAYVKSS TPTTSSFDSE LQKGGNRLDS SDHRGNFSST
ZmPRR1    S--------- --------RP IKTNLRVAES SAFLAYVRSS TPATSSFDSE LQRGGSRLDS LDNQGNCSSA
SbPRR1    S--------- --------RP IKTNLRIAES SAFLAYVKTS TPTTSSLDSE LQRGGSQLDS LDNQGNCSSA
AtPRR1    SS-------- --------AP KKSRLKIGES SAFFTYVKST VLRTNGQDPP LVDGNGSLHL HRGLAEKFQV
HvPRR37   LP-QNNSSDT VMEN-----L EEPIVRAADL IG---SMAKN MDAQQAAARA IDAPNCSLQA PEGKDTD--G
TaPRR37   LP-QNNSNDT VMEN-----S DEPIVRAADL IG---SMAKN MDAQQAAR-A IDAPNCSSQA PQGKDTD--R
BdPRR37   PPSQNNSKET MMEN-----L EEPIVLAADL IG---SMAKN MDAQQAAR-A TDASNSSSKV PEGKDTD--G
OsPRR37   PL-QNNSKEA AMEN-----L EESSVRAADL IG---SMAKN MDAQQAAR-A ANAPNCSSKV PEGKDKN--R
ZmPRR37   PL-QNNSKEA AMEN-----L EESSVRAADL IG---SMAKN MDAQQAAR-A TNAPNCSSKV PEGKDKN--R
SbPRR37   PP-KNNSKES MMQN-----L EDPTVRAADL IG---SMAKN MDTQEAAR-A ADTPNLPSKV PEGKDKNK-H
HvPRR73   ISKNKSVDKI V--------I DKPTSETADL IS---SIARN TESKQAAR-I TDAPDCSSKM PHGNEMNNDS
TaPRR73   TSQNKSVNKI V--------I DKPTSQTADL IS---SIARN TESKQAAR-I TDAPDCSSKM PHGNEMKNDS
BdPRR73   MPQNNSENEV LTESDGINTL DKPAIKTADL IS---SIARN TKAKQAPR-I TDAPDCSSKM PHGNDMNNDS
OsPRR73   MPQSKTTR-- --ETDSRNTQ NEPTTQTVDL IS---SIARS TDDKQVVR-I NNAPDCSSKV PDGNDKNRDS
ZmPRR73   MSKCKSKKET MAEDDCTNMP SATNAETADL IS---SIARN TEGQQAVQ-A VDAPDGPSKM ANGNDKNHDS
SbPRR73   MPQCKSKKKM MAEDDCTDIP SEINTETADL IS---SIARN TEGQQAVR-A VDAPDGPSKM PDGNDKNHDS
AtPRR3    ---------- -------MSM SKKAEEPGDL EKNAKYSVQA LERNNDDTLN RSSGNSQVES ----KAPSSN
AtPRR7    SFEKSSSKWK MKVGKGPLDL SSESPSSKQM HEDGGSSFKA MSSHLQDNRE PEAPNTHLKT LDTNEASVKI
HvPRR59   NACGASKPQV FSAEKNVRSK YLHGITSAKV AGQIMDNAMR IADASSCRPS DLGKDLMATA QPTTSKKCKS
TaPRR59   NACGTSKPQV FSAEKNVHSK YLHGITSAKV AGQIMDNAMR IADASSCRPS DPGKDLMATG QPTTSKKCKS
BdPRR59   NVCETGELQV FSAEKKLRSK CLNGITSAKV AGQIMDNALR IADASSCRPT DPGKDLLAAA PSTAGKKGNS
OsPRR59   NACGDSELQV LSTEKNVRSK FLNGITSAKV AGQIMDNALR FADSSSLRSS DPGKDLLVVA QTTADRKCKS
ZmPRR59   ---------- --SEKNVRSK CLNGITSAKV AEQIMDNALR ITDASSRRPT NLGKDLAMTE P-AADRKCQS
SbPRR59   ---------- --SEKNVHSK CLNGITSAKV AEQIMDNALR ITDASSRRPS NLGKDLAMTE P-EADRKCQS
HvPRR95   ---------- -------RDP ENPSXNKRKK ASTDIEVVHI IDDAPKPST- MEVDIVRTNS KGNGDKWFSI
TaPRR95   ---------- -------RDP ENPSDNKRKK ASTDIEVVHI VDDEPKPSTP MEVDIVRTNS QGNGDKWFSI
BdPRR95   TIRDDNLIPR RERDLSPRKQ PCLKDNDCQK ATREIEVVHI IDDEQKSNAQ TDVDVMRTTF HGNCDKGFSI
OsPRR95   ASNDGNLIPR REEDASLRRM TCSNDINCEK ASRDMELVHI IDNQQKNNTH MEMDVARANS RGNDDKCFSI
ZmPRR95   RAEGNNLIPA REDDLSPKKR TCLNENNSER ASRDMELVHI MENQQKYNTQ WEVDTMRTTS RGNDEKG-SI
SbPRR95   -SKDNNLIPT REDDLSPKKR TCLNDNNSER ASKDMDLVHI MDNQQKHNTQ REVDTMRTTS RGNDEKG-SI
AtPRR5    ---------- ---------- ----ECAKSQ FNETRLLANE LQSKQAEAID FMGASFRRTG RRNREESVAQ
AtPRR9    ---------- ---------- -------RDE FKETFDVTMD LIG----GID KRPDSIYKD- -KSRDECVGP
```

```
                 570        580        590        600        610        620        630
        ....|....| ....|....| ....|....| ....|....| ....|....| ....|....| ....|....|
HvPRR1  TDRID----- ---------- ---------- -----TNSST NIQ----DEK AFEMPTQYPL VCFSSSNLQL
TaPRR1  TDRID----- ---------- ---------- -----TNGKI NIQ----DEK AFETPMQYPL VCFSSSNLHL
BdPRR1  ADRID----- ---------- ---------- -----TNTSI NIE----DEK AFETPMQYPV VCYSSSNSHH
OsPRR1  TDRSD----- ---------- ---------- -----TGTDV NIR----DKE AFEMPVQYPV VCFSSSNLHL
ZmPRR1  TDKSD----- ---------- ---------- -----TGTDV NIR----NKE AFEMPAQYPM VWFSSSNMHM
SbPRR1  TDRSD----- ---------- ---------- -----TVTDV NIR----NKE AFEMPVQYPM VCFSSSNTHM
AtPRR1  VASEG----- ---------- ---------- -----INNTK QARRATPKST VLRTNGQDPP LVNGNGSHHL
HvPRR37 ENAMP----- ---------- ---------- ----YLELSL KRSRSTGEGA G-PIQEEQRN VVRRSDLSAF
TaPRR37 ENAMP----- ---------- ---------- ----YLELSL KRSRSTADGA DAAIQEEQRN VVRRSDLSAF
BdPRR37 DNAMP----- ---------- ---------- ----SLELSL KRPRSTGDGG N-VVQEEPRN VLRRSDLSAF
OsPRR37 DNIMP----- ---------- ---------- ----SLELSL KRSRSTGDGA NAIQE-EQRN VLRRSDLSAF
ZmPRR37 DNIMP----- ---------- ---------- ----SLELSL KRSRSTGDDA NAIQE-EQRN VLRRSDLSAF
SbPRR37 DKILP----- ---------- ---------- ----SLELSL KRSRSCGYGA NTVKADEQQN VLRQSNLSAF
HvPRR73 PINMP----- ---------- ---------- ----SKELGL KISETTR--- RGTEIHDERS ILKRSNLSAF
TaPRR73 PINMP----- ---------- ---------- ----SQELGL KISETAR--- CGTEIHDERS ILKRSNLSAF
BdPRR73 LAIMP----- ---------- ---------- ----SQELGL NISKTTG--- SATEIHEERN VLKRSDLSAF
OsPRR73 LIDMT----- ---------- ---------- ----SEELGL KRLKTTG--- SATEIHDERN ILKRSDLSAF
ZmPRR73 HIEVT----- ---------- ---------- ----PHELGL KRSRTNG--- ATAEIHDERN ILKRSDQSAF
SbPRR73 HIVVT----- ---------- ---------- ----PHELGL KRLRTDG--- AADEIHDERN ILKRSDQSAF
AtPRR3  REDLQ----- ---------- ---------- ----SLEQTL KKTR-----E DRDYKVGDRS VLRHSNLSAF
AtPRR7  SEELM----- ---------- ---------- ----HVEHSS KRHRGTK--D DGTLVRDDRN VLRRSEGSAF
HvPRR59 PITMNNAVKP VMKNTLREDS KGTAIDHPS- -----LDVNL GKQ--QRSDG HVNQELRDKD NFNHSNSSAF
TaPRR59 PVTKNNAVKP VMENTLHENS KGAAIGHPQS CPSHLLDVNL GKQ--QRSDG HVNQELRDKD NFNHSNSSAF
BdPRR59 PAIENSAVNP AMENTPHERS KGTAIGRAES CPPRSLEINL EKQPLFNSNG YANQEFKDKD NFRHSNSSAF
OsPRR59 SALENNAV-- -MENNLSENS KGTATGHAES CPSHFVEINL EKQ--HHLNG YTNHKLNEKD IFNHSNSSAF
ZmPRR59 LVMENNAVK- --EKNPGEKS KSAVIGHADS YPSQFLETNL GKQ--QYRNG YKNQEFREKD IFNHSNSSAF
SbPRR59 SVMENNAVT- --ENNLGVKS KGAAICPADS CPSQFLETNL GKQ--HHLNG YKNQEFREKD IFNHSNSSAF
HvPRR95 PAH------- ---------- ---------- ----QLELSL RRS---DYGR SEDQEKNDTR TLNHSTASAF
TaPRR95 PAH------- ---------- ---------- ----QLELSL RXS---DYGR SEDQEKNDTR TLNHSTSSAF
BdPRR95 PAH------- ---------- ---------- ----QLELSL RRS---DYSK LDDQEKNDKR TLNHSTSSAF
OsPRR95 PAH------- ---------- ---------- ----QLELSL RRS---DYSR LESQEKNERR TLNHSTSSPF
ZmPRR95 PAH------- ---------- ---------- ----QLELSL RRT---DYGK LENHEKNDRR TLNHSTSSAF
SbPRR95 PAH------- ---------- ---------- ----QLELSL RRT---DYGK LENHDKNDRR TLNHSTSSAF
AtPRR5  YESR------ ---------- ---------- ---IELDLSL RRP---NAS- -ENQSSGDRP SLHPSSASAF
AtPRR9  ---------- ---------- ---------- ----ELGLSL KRS---CSVS FENQDESKHQ KLSLSDASAF

                 640        650        660        670        680        690        700
        ....|....| ....|....| ....|....| ....|....| ....|....| ....|....| ....|....|
HvPRR1  EQ-------- ---------- ---------- ---------- ---------- ---RNEGQQD VSGNPPVYHY
TaPRR1  EQ-------- ---------- ---------- ---------- ---------- ---RNEGQQD VSGNPPVYHY
BdPRR1  ER-------- ---------- ---------- ---------- ---------- ---SNQVQHD VSGAPPVYHF
OsPRR1  ER-------- ---------- ---------- ---------- ---------- ---SNEGQND ASGTPPVYHF
ZmPRR1  ER-------- ---------- ---------- ---------- ---------- ---SSEGHND TSGTPPAYHF
SbPRR1  ER-------- ---------- ---------- ---------- ---------- ---SNEGHND TSGTPPVYHF
AtPRR1  HRGAAEKF-- ---------- ---------- ---------- -----QVVAS EGINNTKQAH RSRGTEQYHS
HvPRR37 TRYNMCAVSN QGGAGFVGSC SPNGDSSEAA KT-------- --VAAQMKQG SNGSSNNNDM GSTTKSVVTK
TaPRR37 TRYNTCAVSN QGGAGFVGSC SPNGNSSEAA KT-------- --DAAQMKQG SNGSSNNNDM GSTTKSVVTK
BdPRR37 TRYNTFAVSN QGGTGFVGSC SPHGNSSEAV KTDCTYNVKS SSDAAQMKQG SNGSSNNNDM GSTTKDVVTK
OsPRR37 TRYHTPVASN QGGTGFMGSC SLHDNSSEAM KTDSAYNMKS NSDAAPIKQG SNGSSNNNDM GSTTKNVVTK
ZmPRR37 TRYHTPVASN QGGTGFVGSC SPHDNISEAM KTDSAYNMKS NSDAAPIKQG SNGSSNNNDM GSTTKNVVTK
SbPRR37 T--------- ---------- -----SSEAM KTDSTYNMKS NSDAAPIKQG SNGSSNNNDM GSTTKNVVTK
HvPRR73 TRYHTPMASD QGGATFRGSC SPQDNSSEAV KTNSTCKMES NSDAAQIKQG SNGSSNNNDM GSSTKNANTK
TaPRR73 TRYHTPMASD QGGATFRGSC SPQDNSSEAV KTNSTCKMES NSDAAQIKQG SNGSSNNNDM GSSTKNAIAK
BdPRR73 TRYHTSMVSD QGGARFRGSC SPQDNSSEAV KTDSTFKMKS DSDAAPIKQG SNGSSNNNDM GSSTKNAMTT
OsPRR73 TRYHTTVASN QGGAGFGGSC SPQDNSSEAL KTDSNCKVKS NSDAAEIKQG SNGSSNNNDM GSSTKNAITK
ZmPRR73 TRYHTSVASN QGGARYGESS SPQDNSSEAM KTDSTCKMKS NSDAAPIKQG SNGSSNN-DV GSSTKNVAAR
SbPRR73 TRYHTSVASN QGGARCGESS SPQDNSSEAV KTDSTCKMKS NSDAAPIKQG SNGSSNN-DV GSSTKNVIAK
AtPRR3  SKYNN----- ---------- ---------- ---------G ATSAKKAPEE NVESCSPHDS PIAKLLG---
AtPRR7  SRYN------ ---------- ---------- ---------P ASNANKISGG NLGSTSLQDN NSQDLIKKTE
HvPRR59 SRYGNKRIEP SAKQLSLPSV HLTYQE-SVN DKN--VQSSG VLPS-HEHHT CKITMQAQAP LDSCTEGPAI
TaPRR59 SRYGNKRIEP SAKQLSLPSV HLTHQE-SVY DKN--VQSSG TLPS-HEHNT CKITMQAQAP LDSCTVGPAI
BdPRR59 SRYGNKRIES SVQQLFPPSL HLSHHE-PVC DKN--IQPGG ALSS-REHNT WKSAVQAKVP LDSCTERVAI
OsPRR59 SRYGNKRIES SAQRPFPPSF RVVHQQ-PVY DKN--PQSSR VLLS-CEHNT RESTVQAQVP LDRSTEGAAI
ZmPRR59 SRYGNRRIES SGEIQFLPSR CIVGQEQHVH GKDPVFQPNG VLLPPNDHNT GESTRQARIT LDSSMEGADI
SbPRR59 SRYGNKRIEP SGEIQFFPSL CITGQE-HVH GKDPVFQPNG VLLPPNEHNT GESTRQTRIT LDSSPEGAAI
HvPRR95 SLYNCRPVSS FGNAGDAQPC STSATH---- ---------- ---VDPANKN GNWAAPSQDK THTXCHPIRV
TaPRR95 SLYNCRPISS FGNAGDAQPC STSATH---- ---------- ---ADLANKN GDSAAPFQDK ADPICHPIRV
BdPRR95 SLYNCRTASS CGNAGDAHLC STSATH---- ---------- ---VDLEMKT GDSVAPSQDK TDAICPPIRV
OsPRR95 SLYNCRTASS TINAGDAQAC STSATH---- ---------- ---IDLENKN GDSKTPSQDK RETNQPPIRV
ZmPRR95 SLYNCRAVPT LGNAGDGQLC STSETL---- ---------- ---VDVENKN GDSADPSQDM TETNRPPIRV
SbPRR95 SLYNCRAVST SGNAGDGQLC STSETL---- ---------- ---MDVENKN GDSAAPSQDM TETNRPPIRV
AtPRR5  TRYVHRPLQT QCSAS----- PVVTDQ---- ---------- ---RKNVAAS QDDNIVLMNQ YNTSEP----
AtPRR9  SRFEE----- ---------- ---------- ---------- -------SKS AEKAVVALEE STSGEP----
```

```
                  710       720       730       740       750       760       770
          ....|....| ....|....| ....|....| ....|....| ....|....| ....|....| ....|....|
HvPRR1    P-------FY YPG------- ----MVEHSM ALHS-----V QSFQ-GNINT AQAHT----- ----------
TaPRR1    P-------FY YPG------- ----MVEHGM TLHS-----V QSFQ-GNINT AQAHT----- ----------
BdPRR1    P-------FY YPG------- ----MIEHGM ALPP-----V QNFQ-GNINS AQPHT----- ----------
OsPRR1    P-------FY YPG------- ----MMDHGM THPP-----V QNFQ-GNINN AQVHT----- ----------
ZmPRR1    P-------FY YPG------- ----MVEHNM ALSS-----A QDFQ-ANINN AQAHT----- ----------
SbPRR1    P-------FY YPG------- ----MVEHNM AVSS-----V QNFQ-ANINN AQAHT----- ----------
AtPRR1    QGETLQNGAS YPH------- ----SLERSR TLPTSMESHG RNYQEGNMNI PQVAMNRSKD SSQVDGSGFS
HvPRR37   PCG--NNKVS PIN-----GN THTSAFHRVQ PWTP---ATA AGKDKADEVS KKNVAAAAAA AKEMGCEAQS
TaPRR37   PAGG-NNKVS PIN-----GN THTSAFHRVQ PWTP---ATA AGKDKADEMS KKN-AATAAA AKDNGGEAQS
BdPRR37   PGS---NKFS AIN-----GS THTSAFHRVQ QWTP---AVA G----KDGVG KKN-AATTAG KDDKGGEAES
OsPRR37   PSTN-KERVM SPS--AVKAN GHTSAFHPAQ HWTS-PANTT G-KEKTDEVA NNAAKRAQPG EVQSNLVQ-H
ZmPRR37   PSTN-KERVM SPS--AVKAN GHTSAFHPAQ HWTS-PANTT G-KEKTDEVA NNAAKRAQPG EVQSNLVQ-H
SbPRR37   PTTNNKDRVM LPSSAINKAN GHTSAFHPVQ HWTMVPANAA GGTAKADEVA NIAGYPS--G DMQCNLMQWY
HvPRR73   PCTDRERVMP PSL---VKSN QQTSAFHPVQ HQVS--PADA VGKDKAAEEI ANAVKVDHSS EAQQSSVQHH
TaPRR73   PCTDRERVMS PSL---VKSN QQTSAFHPVQ HQVS--PADA ARKDKASEEI VNAVKVGHSS EAQQSSVQHH
BdPRR73   PSADR-GPLS PSA---IKSN HYTSAFHPVQ YQTS--PPNV LRKDKAEEET VNVVKVDYSR EAQQSSVQHH
OsPRR73   PSSNRGKVIS PSA---VKAT QHTSAFHPVQ RQTS--PANV VGKDKVDEGI ANGVNVGHPV DVQNSFMQHH
ZmPRR73   PSGDRERVAS PLA---IKST QHASAFHTIQ NQTS--PANL IGEDKADEGI SNTVKMSHPT EVPQGCVQHH
SbPRR73   PSANRERVTS PSA---IKST QHASAFHTIQ NQTS--PANL VGKDKADEGI SNAVKMSHPT EVPQSCVQHH
AtPRR3    ---------- ---------- --------SS SSSD---NPL KQQ------S SGS------- ----------
AtPRR7    AAYDCHSNMN ESLPHNHRSH VGSNNFDMSS TTEN---NAF TKPGAPKVSS AGSSSVKHSS FQPLPCDHHN
HvPRR59   PSSTSAREDA GTS------- --SSSPRKDN FGHPPYGFIP VPIPVGAAIP YHYSAIMPPI YYTQPPFMQC
TaPRR59   PSSSSAREDA GTS------- --SSSPRKDN FGHPPYGFIP VPISVAAAIP YQYSAIMPPI YYTQPPFMQC
BdPRR59   LSSSSAREDA GPS------- --SSSPRTEI LNHPPYGFIP VPIPVGAAIP YHYGAIMQPI YYPQAPFMQH
OsPRR59   LCSSSVREDA GTS------- --SSSPRKDS LTHPSYGFIP VPIPVGAAIP YHYGAIMQPM YYPQGAFMHC
ZmPRR59   ICSSSAREDA GAS------- --SSSHRKDS MSHPSYGFVP VPIPVGAMMP YHCGAILQPV YYPQGPLMHC
SbPRR59   MCSSSAREDA GVS------- --SSSHRKDS MSHPSYGFIP VPIPVGPGMP YHYGAILQPV YYPQGPLMHC
HvPRR95   VPLPAPVGGL TFD------- --GQPFWSGA PVAPLLYPQS GPPIWNSRTP VSQKADTQAT SSQQKCQQND
TaPRR95   VALPVPVGGL TFD------- --GXPFWSGA PVAPLLYPQS GPPIWNGRTP VSQEADTQAT SSQQKCQQND
BdPRR95   VPFPVPVAGL TFN------- --GQPFWSGA PVAPLLYPQS GPPIWNSKTS TSKQAAAQAI LSQQKWQQSN
OsPRR95   VPFPVPVGGL TFD------- --GQPFWNGA PVASLFYPQS APPIWNSKTS TWQDATTQAI SLQQ----NG
ZmPRR95   VP--VPVQGL TFD------- --GQPFWNGT PVASLFYSQS TPPIWNSKTS MWQESTPQAT SLPQKSPQNE
SbPRR95   VPFPVPVQGL TFD------- --GQPFWNGT PMASLFYPQS APPIWNSKTS MWQEST-QAT SLPQKSQQNE
AtPRR5    -----PPNAP RRN------- --DTSFYTGA --------DS PGPPFSNQLN SWPGQSSYPT PTPINNIQFR
AtPRR9    -----KTPTE SHE------- --KLRKVTSD --------QG SATTSSNQEN --IGSSS--- ------VSFR

                  780       790       800       810       820       830       840
          ....|....| ....|....| ....|....| ....|....| ....|....| ....|....| ....|....|
HvPRR1    ---------- ---------- ------PPTM LHQYS---VY HQSHGASTMP SYQYNPAGMN VHSS--HLSM
TaPRR1    ---------- ---------- ------PPTM LHQYN---VY HQSHGAS-MQ SYQYSPAGMN VHSS--HLST
BdPRR1    ---------- ---------- ------PPTL LHQYN---VY PQSHGVSMMP SFQYNHAGMS FHQSS-HLSM
OsPRR1    ---------- ---------- ------PQTL LPQYN---VY PQCHGVSMMP PFQYNPAGMS IQSN--QLPT
ZmPRR1    ---------- ---------- ------PPTM LPQYN---VY PQCHGLPVIP SFQFNPSGMS THSS--HLPT
SbPRR1    ---------- ---------- ------PPTM LPQYN---VY PQCHGLPMIS SFQFNPAGMN MHSS--HLPT
AtPRR1    APNAYPYYMH GVMNQ----- --VMMQSAAM MPQYGHQIPH CQPNHPNGMT GYPYYHHPMN TSLQHSQMSL
HvPRR37   KHPCA----- AADDV----- --NGGS-AGG TAQSSVVNPS GPVEGHAANY G---SNSFSN NNTNN---GS
TaPRR37   KRPCA----- AAHDA----- --NGGSSAGG TAQSNVVNPS GPVEGHAANY G---SNSGSN NNTNN---GS
BdPRR37   KQPPR----- AAHDD----- --QENGGSAE GLQSNVTDPS APLEGHAANY G---SNSGSN NNNNNNNNGS
OsPRR37   PRPILHYVHF DVSRE----- --NGGSGAPQ CGSSNVFDP- -PVEGHAANY GVNGSNSGSN NGSNGQNGST
ZmPRR37   PRPILHYVHF DVSRE----- --NGGSGAPQ CGSSNVFDP- -PVEGHAANY GVNGSNSGSN NGSNGQNGST
SbPRR37   PRPTLHYVQF DGARE----- --NGGSGALE CGSSNVFDP- -PVEGQATNY GVNRSNSGSN NATKGQNGSN
HvPRR73   HHGHYYRHVM VQQQ-----T LIDR-ASNAR CGSSNASDS- -PMEGHAANY GVNGSISGSN NGSNTQNRSS
TaPRR73   HHAHYYRHVI AQQQ-----T LIDR-ASNAR CGSSNASDL- -PLEGHAANY GVNGSISGSN NGSNTQNASS
BdPRR73   HHVHYYLHVM SQQQ-----P SIDR-ASDAR CGLSNVSDP- -PIEGHAANY GVNGSISGSH NGSNVQNESS
OsPRR73   HHVHYYVHVM TQQQQQ---P SIERGSSDAQ CGSSNVFDP- -PIEGHAANY SVNGSFSGGH NGNNGQRGPS
ZmPRR73   HHVHYYLHVM TQKQ-----P STDRGSSDVH CGSSNVFDP- -PVEGHAANY SVNGGVSVGH NGCNGQNGSS
SbPRR73   HHVHYYLHVM TQKQ-----S SIDRGSSDVQ CGSSNVFDP- -PVEGHAANY SVNGGVSVGH NGCNGQNGTS
AtPRR3    ---------- ---------- ---------- ---------- ---------- ---------- ----------
AtPRR7    NHASYNLVHV AERKK----- ------LPPQ CGSSNVYNET IEGNNNTVNY SVNGSVSGSG HGSNGPYGSS
HvPRR59   DPSGINQMGI QHA-YHSSYH QNLS--KPSE IDEHRQLEEN QRLHH-SRQI LQESGEPIDL LRAHAEHNNQ
TaPRR59   DPSGINQMGI QHA-YHSSYH QNLG--KPSE IDEHRQLEEN QRLHH-SKQI LQESGEPIDL LRAHAERNNQ
BdPRR59   DPSAINQMAI QHASFHSNYH QSLG--KPSE VVEHRQLEEN QLLHHHSRKI LRES-EPIDL SRP--ENANP
OsPRR59   DSAAINKTAI QHVSCQSNYH ENLG--KPPQ IDEHKQPEEN HQLHH-SRQI LRESGEPVDL AKAHMERINQ
ZmPRR59   NSSAINKATI HQTSGQSNYR EDCGSGRPSQ VDEHKQSEEK HQLHH-SRQI HRESGQPVDM VRAHMDHANQ
SbPRR59   DSAGINKAAI QHGSGQSNYH EAPG--KPSQ DDEHKQSEEN HQLHH-SRQI LRESGEPIEM ARAHMDRANQ
HvPRR95   PTEMDCQQTE STQ------- --LQEVLPPP IANEKHLHVE IPSDSNTQQV SPMTGESGS- -STVLNNSGN
TaPRR95   PTEMDSQQTE STQ------- --QQEVLPPP TANEKHLHVE IPSDSNPQQV SPMAGDSGSG SSTVLNNSG-
BdPRR95   ATVMDSDQAE ITQ------- --GQEVLPAP NANEKHLHVE IPSD-DPQHV SPMTGDSGS- -STVLNNSG-
OsPRR95   PKDTDTKQVE NVE------- --EQTARSHL SANRKHLRIE IPTD-EPRHV SPTTGESGS- -STVLDSAR-
ZmPRR95   PNEMGAKPVI NAG------- --EQFAMGPP SASGKQLHVE ILND-DPRHI SPMTGESGI- -STVLDSTR-
SbPRR95   PNEMGAKPVE NAE------- --EQFVTGPP SASGKQLCVE VPKD-DPRHI SPMTGESGI- -STMLDSTR-
AtPRR5    DPNTAYTSAM APA------- --SLSPSPSS VSPHEYSSMF HPFNSKPEGL QDRDCSMDVD ERRYVSSATE
AtPRR9    --NQVLQSTV TNQ------- --KQD-SP-- ---------- IPVESNRE-- --KAASKEVE AG----SQST
```

```
                  850        860        870        880        890        900        910
         ....|....| ....|....| ....|....| ....|....| ....|....| ....|....| ....|....|
                                                                                  °°°°°°
HvPRR1   QN-------- ---------- ----VWSSVS STP-ISEERH GHSG------ ---------- ---RRAAALA
TaPRR1   QN-------- ---------- ----VWSSVS STP-ILEERH SRSG------ ---------- ---RRAAALA
BdPRR1   QN-------- ---------- ----VWSSVS STP-VPDETC SRSD------ ---------- ---RRAAALA
OsPRR1   QN-------- ---------- ----MWPQAS STP-MPEETC SRSE------ ---------- ---RRAAALA
ZmPRR1   QN-------- ---------- ----VWSSAS STPPVPEETC SRSE------ ---------- ---RRAAALA
SbPRR1   QN-------- ---------- ----VWSSAS STP-MPEETC NRSE------ ---------- ---RRAAALA
AtPRR1   QNGQMS---- ---------- MVHHSWSPAG NPP-SNEVRV NKLD------ ---------- ---RREEALL
HvPRR37  TAATT----- ---------- -------AAA ATVHAETGGG VDKR----SN -MMNMK---- -RERRVAAVN
TaPRR37  TAAT------ ---------- --------AA AAVHAETGG- IDKR----SN -MMHMK---- -RERRVAAVN
BdPRR37  TAAAV----- ---------- -------AAS TTAVNVEAGG IDKR----SS SIMYMK---- -RELRVAAVT
OsPRR37  TA-------- ----VNAERP NMEIANGTIN KSG-PGGGNG SGSG----SG NDMYLKRFTQ -REHRVAAVI
ZmPRR37  TA-------- ----VNAERP NMEIANGTIN KSG-PGGGNG SGSG----SG NDMYLKRFTQ -REHRVAAVI
SbPRR37  TVGASMAGPN ANANGNAGRT NMEIANEVID KSGHAGGGNG SGSG----SG NDTYVKRLAA GLTPRQAQLK
HvPRR73  FAPNIA---- --------RP NMES--GTMD KTE-AGGGNG SGSGPSGSGT DLVCQN---- QLSQREAAVN
TaPRR73  SAPNIA---- --------RP NMDS--GAMD KTE-AGGGTG SGSGPSGSGN DMVCQN---- QLSQREAAVN
BdPRR73  TAPNVT---- --------RP NMES--GIMD ENG-AEGGNG SGSGPSGGSG NDMYQN---- QLSQREAAVN
OsPRR73  TAPNVG---- --------RP NMETVNGIVD ENG-AGGGNG SGSG----SG NDLYQN---- GVCYREAALN
ZmPRR73  AVPNIA---- --------RP NIESINGTMS QNI-AGGGIV SGSG----SG NDMYQN---- RFLQREAALN
SbPRR73  TVPNIA---- --------RP NIESVNGTVS QNI-AGGGIV SGSG----SG NDVYQN---- RFPQREAALN
AtPRR3   ---------- ---------- ---------- ---------- ---------- -DRWAQ---- ----REAALM
AtPRR7   NGMNAG---- --------GM NMGSDNGAGK NGNGDGSGSG SGSGSGNLAD ENKISQ---- ----REAALT
HvPRR59  SASCSQ---- --------DI R---GWTVSG ETDTNTNTVI APESGNESGI QNFGYNGLDS DRSRREAALM
TaPRR59  SASCSQ---- --------DI R---GWTVSG ETDTNTNAII APESGNESGI QNFGYNGLDS DRSRREAALM
BdPRR59  STSCSQ---- --------DL RRGSGWTVSG ETDMNTNTII AMESGNDSGI QNFSNNGLDI DRSRREAALM
OsPRR59  SASCSQ---- --------DI RKGSGCTGSG ETDANTNTVI ALESGNESGV QNCSNNVLDG DRSRREAALL
ZmPRR59  SASCSR---- --------DI CKGSGCTGSG EADTNANTTV ALESGNESGI Q-------NS DRSHREAALM
SbPRR59  SASCSQ---- --------DI CKGSGCTGSG EADINANTMV ALESGNESGI Q-------NG DRSRREAALM
HvPRR95  SNALSG---- --------SA CGSSSNRITT PTEQCNASDG ATENPSMEGL HH-------- -LSQREIALN
TaPRR95  -NALSG---- --------SA CGSSSNRIAT PTEQCNASDG ATENPSMEGS HQ-------- -LSQREIALN
BdPRR95  -NAPSG---- --------SG CDSSSNRIVA PLDPCNSFNG VPENPSMDGT HH-------- -LSQREVALN
OsPRR95  -KTLSG---- --------SV CDSSSNHMIA PTESSNV--- VPENP--DGL RH-------- -LSQREAALN
ZmPRR95  -NTLSS---- --------SG CDSISNQITA PTESSNVYKD VPETPSAEGS RH-------- -LSQREAALN
SbPRR95  -NTLSS---- --------SG CDSTSNQFTA PTESSNVYKG VPETPSTEGS RH-------- -LSQREAALN
AtPRR5   HSAIGN---- --------HI DQLIEKKNED GY-------- ---SLSVGKI QQ-------- -SLQREAALT
AtPRR9   NEGIAG---- --------QS SSTEKPKEEE ---------- -----SAKQR WS-------- -RSQREAALM

                 920        930        940        950        960        970        980
         ....|....| ....|....| ....|....| ....|....| ....|....| ....|....| ....|....|
         °°°°°°°°°°°°°°°°°°°°°°°°°°°°°°°°°°°°°°°°

HvPRR1   KFRQKRKDRC FDKKVRYVNR KKLAETRPRV RGQFVRQASN TSYTDIISTG D--DISEYED DDPSSRDVEL
TaPRR1   KFRQKRKDRC FDKKVRYVNR KKVAETRPRV RGQFVRQASN ---TDIISTG D--DISEYED DDPSSRDVEL
BdPRR1   KFRQKRKERC FDKKVRYVNR KKLAEIRPRV RGQFVRQAGN ---ADIISTG D--DISEDED D-PSSREVEM
OsPRR1   KFRLKRKERC FDKKVRYVNR KKLAETRPRV RGQFVRQANY ---TDITSTG D--DISEDED DDPSSREVEM
ZmPRR1   KFRLKRKERC FDKKVRYVNR KKLAETRARV RGQFVRQPTN ---TDVISIG D--DISEDED DDPSSREVDM
SbPRR1   KFRQKRKERC FDKKVRYVNR KKLAETRPRV RGQFVRQASN ---TDITSTG D--DISEDED DDPSSREVDI
AtPRR1   KFRRKRNQRC FDKKIRYVNR KRLAERRPRV KGQFVRKMNG -VNVDLNGQP DSADYDDEEE EEEEEEEENR
HvPRR37  KFREKRKERN FGKKVRYQSR KRLAEQRPRV RGQFVRQPP- ---------- -----APAAV ER--------
TaPRR37  KFREKRKERN FGKKVRYQSR KRLAEQRPRV RGQFVRLPL- ---------- -----RDNLP PHT-------
BdPRR37  KFREKRKERN FGKKVRYQSR KRLAEQRPRV RGQFVRQPA- ---------- -----VAAAT ER--------
OsPRR37  KFRQKRKERN FGKKVRYQSR KRLAEQRPRV RGQFVRQAV- ---------- -----QDQQQ QGGGREAAAD
ZmPRR37  KFRQKRKERN FGKKVRYQSR KRLAEQRPRV RGQFVRQAV- ---------- -----QDQQQ QGGGREAAAD
SbPRR37  KYREKKKDRN FGKKVRYQSR KRLADQRPRF RGQFVKQAL- ---------- -----QDQGE QDGTGER---
HvPRR73  KFRQKRKERN FGKKVRYQSR KRLAEQRPRV RGQFVRQSG- ---------- -----QEDEA GQAADR----
TaPRR73  KFRQKRKERN FGKKVRYQSR KRLAEQRPRV RGQFVRQSG- ---------- -----QEDEA GQAEDR----
BdPRR73  KFRQKRKERN FGKKVLYQSR KRLAEQRPRV RGQFVKQSG- ---------- -----QEDQA GQAADR----
OsPRR73  KFRQKRKVRN FGKKVRYQSR KRLAEQRPRI RGQFVRQSG- ---------- -----QEDQA GQDEDR----
ZmPRR73  KFRLKRKDRN FGKKVRYQSR KRLAEQRPRV RGQFVRQSE- ---------- -----QEDQT AQGSER----
SbPRR73  KFRLKRKDRN FGKKVRYQSR KRLAEQRPRV RGQFVRQSG- ---------- -----QEDQA AQGSER----
AtPRR3   KFRLKRKERC FEKKVRYHSR KKLAEQRPHV KGQFIRK--- ---------- -----RDDHK SGSEDN----
AtPRR7   KFRQKRKERC FRKKVRYQSR KKLAEQRPRV RGQFVRKTAA AT-------- -----DDNDI KNIEDS----
HvPRR59  KFRMKRKDRC FEKKVRYHSR KKLAEQRPRI KGQFVSQKLK S--------- -----ATTTE DAETD-----
TaPRR59  KFRMKRKDRC FEKKVRYHSR KKLAEQRPRI KGQFVSQKLK S--------- -----ATTTE DAETD-----
BdPRR59  KFRMKRKDRC YEKKVRYHSR KKLAEQRPRI KGQFVSQKLK SD-------- --TATPTTTE DVETD-----
OsPRR59  KFRMKRKDRC FEKKVRYHSR KKLAEQRPRV KGQFVSQKLK S--------- ------AITT EAETD-----
ZmPRR59  KFRMKRKDRC FDKKVRYHSR KKLAEQRPRV KGQFVSQKLK A--------- ------AMAT EAETDS----
SbPRR59  KFRMKRKDRC FEKKVRYHSR KKLAEQRPRV KGQFVSQKQK S--------- ------ATTT EAETDL----
HvPRR95  KFRLKRKERC FEKKVRYQSR KLLAEQRPRV KGQFVRQ--- ---------- ------DQSV QGSS------
TaPRR95  KFRLKRKERC FEKKVRYQSR KLLAEQRPRV KGQFVRQ--- ---------- ------EQNI QAR-------
BdPRR95  KFRLKRKERC FEKKVRYQSR KLLAEQRPRV KGQFVRQ--- ---------- ------EPSI QGS-------
OsPRR95  KFRLKRKDRC FEKKVRYQSR KLLAEQRPRV KGQFVRQ--- ---------- ------DHGV QGS-------
ZmPRR95  KFRLKRKDRC FEKKVRYQSR KLLAEQRPRV KGQFVRQ--- ---------- ------DHSI QGS-------
SbPRR95  KFRLKRKDRC FEKKVRYQSR KLLAEQRPRV KGQFVRQ--- ---------- ------DHSI QGS-------
```

```
AtPRR5   KFRMKRKDRC YEKKVRYESR KKLAEQRPRI KGQFVRQ--- ---------- ------VQST QAP-------
AtPRR9   KFRLKRKDRC FDKKVRYQSR KKLAEQRPRV KGQFVRT--- ---------- ------VNSD ASTKS-----


                 990
         ....|....| .
HvPRR1   VSSPE----- -
TaPRR1   VSSPE----- -
BdPRR1   VSSPE----- -
OsPRR1   VSSPE----- -
ZmPRR1   VSSPE----- -
SbPRR1   VSSPE----- -
AtPRR1   DSSPQDDALG T
HvPRR37  ---------- -
TaPRR37  ---------- -
BdPRR37  ---------- -
OsPRR37  R--------- -
ZmPRR37  R--------- -
SbPRR37  ---------- -
HvPRR73  ---------- -
TaPRR73  ---------- -
BdPRR73  ---------- -
OsPRR73  ---------- -
ZmPRR73  ---------- -
SbPRR73  ---------- -
AtPRR3   ---------- -
AtPRR7   ---------- -
HvPRR59  ---------- -
TaPRR59  ---------- -
BdPRR59  ---------- -
OsPRR59  ---------- -
ZmPRR59  ---------- -
SbPRR59  ---------- -
HvPRR95  ---------- -
TaPRR95  ---------- -
BdPRR95  ---------- -
OsPRR95  ---------- -
ZmPRR95  ---------- -
SbPRR95  ---------- -
AtPRR5   ---------- -
AtPRR9   ---------- -
```
