# Supplementary material for: Expression conservation within the circadian clock of a monocot: natural variation at barley Ppd-H1 affects circadian expression of flowering time genes, but not clock orthologs
Source: BMC Plant Biol. 2012 Jun 21;12:97. doi: 10.1186/1471-2229-12-97 (PMC3478166; doi:10.1186/1471-2229-12-97)
Supplement: Additional file 5 — Estimates of average genetic distance between GIGANTEA (a), CCA1/LHY (b) and PRRs (c) sequences. [file 1471-2229-12-97-S5.pdf]

Supplementary Table 2: Estimates of average genetic distance between GIGANTEA (a), CCA1/LHY (b) and PRRs (c) sequences.

(a)	HvGI	TaGI	BdGI	OsGI	ZmGI	SbGI	AtGI
HvGI							
TaGI	[illegible]						
BdGI	0.067	[illegible]					
OsGI	0.111	0.123	[illegible]				
ZmGI	0.126	0.139	0.113	[illegible]			
SbGI	0.128	0.139	0.111	0.085	[illegible]		
AtGI	0.383	0.378	0.373	0.364	0.373	[illegible]	

(b)	AtCCA1	AtLHY	BdCCA1	HvCCA1	OsCCA1	SbCCA1	TaCCA1	ZmCCA1
AtCCA1								
AtLHY	[illegible]							
BdCCA1	0.922	[illegible]						
HvCCA1	0.936	0.927	[illegible]					
OsCCA1	0.909	0.914	0.251	[illegible]				
SbCCA1	0.909	0.905	0.258	0.292	[illegible]			
TaCCA1	0.914	0.922	0.189	0.044	0.256	[illegible]		
ZmCCA1	0.940	0.922	0.283	0.302	0.254	0.097	[illegible]	

(c)	AtPRR1	AtPRR3	AtPRR5	AtPRR7	AtPRR9	HvPRR1	HvPRR37	HvPRR73	HvPRR59
AtPRR1									
AtPRR3	1.1802								
AtPRR5	1.2618	1.0866							
AtPRR7	1.2202	0.6011	1.0515						
AtPRR9	1.2408	0.9376	0.7945	1.0176					
HvPRR1	0.7175	1.1607	1.1607	1.1047	1.1047				
HvPRR37	1.2202	0.7053	1.0866	0.5902	1.0515	1.0689			
HvPRR73	1.3053	0.7175	1.1047	0.6234	1.0515	1.0866	0.3329		
HvPRR59	1.1802	0.9531	0.9376	1.0866	0.9223	1.1802	1.0011	0.9223	
HvPRR95	1.2408	0.9531	0.8079	0.9849	0.8779	1.2618	0.9689	1.0176	0.8925
TaPRR1	0.7425	1.2000	1.1802	1.1802	1.1607	0.0881	1.1230	1.1230	1.2202
TaPRR37	1.2408	0.6931	1.1230	0.5794	1.0345	1.1047	0.0621	0.3245	1.0011
TaPRR73	1.2618	0.6931	1.1047	0.6234	1.0689	1.0689	0.3162	0.0368	0.9073
TaPRR59	1.2000	0.9531	0.9376	1.0866	0.9223	1.2000	1.0176	0.9376	0.0244
TaPRR95	1.2202	0.9689	0.8635	0.9849	0.8925	1.2202	1.0011	1.0011	0.8779
BdPRR1	0.7175	1.1047	1.1230	1.0689	1.1047	0.2216	1.0515	1.0866	1.2000
BdPRR37	1.2833	0.7053	1.0176	0.5794	1.0176	1.1047	0.1562	0.3413	0.9689
BdPRR73	1.3278	0.7425	1.1802	0.6693	1.0689	1.0345	0.3162	0.1353	0.9689
BdPRR59	1.2202	0.9376	0.9531	1.0515	0.9376	1.1802	0.9689	0.9073	0.1284
BdPRR95	1.2408	0.9376	0.8493	0.9531	0.9223	1.2000	0.9689	0.9849	0.8635
ZmPRR1	0.7553	1.0866	1.1802	1.1047	1.1047	0.2216	1.0176	1.0345	1.2202
ZmPRR37	1.2618	0.6812	1.0176	0.5581	1.0011	1.0515	0.1775	0.2757	0.9223
ZmPRR73	1.2408	0.7175	1.0515	0.6812	0.9376	1.0515	0.3846	0.1993	0.9223
ZmPRR59	1.1802	0.9376	0.9849	1.0515	0.9223	1.1607	0.9531	0.8779	0.2216
ZmPRR95	1.2000	0.8779	0.8079	0.8635	0.9073	1.2000	0.9376	0.9376	0.8925
SbPRR1	0.7300	1.0866	1.1802	1.1047	1.1230	0.1993	1.0689	1.0515	1.1802
SbPRR37	1.3984	0.7945	1.1417	0.6693	1.1047	1.1802	0.3162	0.4025	0.9849
SbPRR73	1.2408	0.6812	1.0689	0.6693	0.9531	1.0345	0.3935	0.1703	0.8925
SbPRR59	1.2202	0.9376	0.9531	1.0515	0.9223	1.1607	0.9689	0.8779	0.1703
SbPRR95	1.2000	0.8925	0.7682	0.8925	0.8779	1.2000	0.9223	0.9689	0.8925
OsPRR1	0.7175	1.1047	1.1417	1.1230	1.1047	0.1775	1.0689	1.0866	1.2000
OsPRR37	1.2618	0.6812	1.0176	0.5687	1.0011	1.0515	0.1847	0.2917	0.9376
OsPRR73	1.2833	0.7300	1.0866	0.6461	1.0345	1.0689	0.3584	0.1491	0.8779
OsPRR59	1.1607	0.9223	1.0176	1.0176	0.9531	1.1802	0.9376	0.8635	0.2216
OsPRR95	1.2408	0.9689	0.8353	0.9689	0.9223	1.1802	0.9376	1.0011	0.8925

(c)	HvPRR95	TaPRR1	TaPRR37	TaPRR73	TaPRR59	TaPRR95	BdPRR1	BdPRR37	BdPRR73
HvPRR95									
TaPRR1	1.2833								
TaPRR37	0.9849	1.1417							
TaPRR73	1.0011	1.1047	0.3080						
TaPRR59	0.8925	1.2408	1.0176	0.9223					
TaPRR95	0.0750	1.2408	1.0176	0.9849	0.8779				
BdPRR1	1.2000	0.1993	1.0866	1.0689	1.2000	1.1607			
BdPRR37	0.9531	1.1417	0.1775	0.3329	0.9849	0.9689	1.1047		
BdPRR73	1.0011	1.0689	0.3080	0.1284	0.9689	0.9849	1.0515	0.3245	
BdPRR59	0.8925	1.2000	0.9689	0.8925	0.1353	0.8779	1.1607	0.9376	0.9689
BdPRR95	0.2522	1.2202	0.9849	0.9689	0.8635	0.2445	1.1047	0.9531	0.9689
ZmPRR1	1.2408	0.2216	1.0515	1.0176	1.2408	1.2202	0.2522	1.0345	1.0345
ZmPRR37	0.8635	1.0866	0.1562	0.2757	0.9376	0.8925	1.0689	0.1847	0.2522
ZmPRR73	0.9531	1.0866	0.3758	0.1993	0.9376	0.9376	1.0689	0.3935	0.2141
ZmPRR59	0.9073	1.2000	0.9849	0.8635	0.2445	0.8925	1.1607	0.8925	0.9376
ZmPRR95	0.3162	1.2202	0.9376	0.9223	0.8925	0.3413	1.1607	0.9223	0.9689
SbPRR1	1.2618	0.1920	1.1047	1.0345	1.2000	1.2408	0.1993	1.0866	1.0345
SbPRR37	0.9849	1.2202	0.2998	0.4025	1.0011	1.0176	1.2000	0.2917	0.3935
SbPRR73	0.9531	1.0689	0.3846	0.1775	0.9073	0.9376	1.0689	0.3935	0.1993
SbPRR59	0.8779	1.2000	0.9849	0.8635	0.1920	0.8635	1.1607	0.9073	0.9376
SbPRR95	0.2917	1.2202	0.9223	0.9376	0.8925	0.3162	1.1607	0.9223	0.9849
OsPRR1	1.2202	0.1703	1.1047	1.0689	1.2202	1.1607	0.1775	1.1047	1.0345
OsPRR37	0.8635	1.0866	0.1632	0.2917	0.9531	0.8925	1.0689	0.1993	0.2678
OsPRR73	0.9531	1.1047	0.3498	0.1491	0.8925	0.9376	1.0515	0.3584	0.1562
OsPRR59	0.9073	1.2202	0.9531	0.8493	0.2292	0.8779	1.1802	0.8779	0.9223
OsPRR95	0.2678	1.2000	0.9531	0.9689	0.8925	0.2917	1.1230	0.9689	0.9849

(c)	BdPRR59	BdPRR95	ZmPRR1	ZmPRR37	ZmPRR73	ZmPRR59	ZmPRR95	SbPRR1	SbPRR37
BdPRR59									
BdPRR95	0.8925								
ZmPRR1	1.2000	1.2000							
ZmPRR37	0.8925	0.8925	1.0345						
ZmPRR73	0.9073	0.9376	1.0176	0.3245					
ZmPRR59	0.2757	0.8779	1.1802	0.9223	0.8635				
ZmPRR95	0.9073	0.3162	1.2202	0.8779	0.8925	0.9223			
SbPRR1	1.1607	1.2000	0.0815	1.0515	1.0515	1.1417	1.2408		
SbPRR37	0.9531	1.0011	1.1607	0.2292	0.4206	1.0176	0.9689	1.1802	
SbPRR73	0.8779	0.9376	1.0011	0.3162	0.0494	0.8493	0.8925	1.0345	0.4115
SbPRR59	0.2141	0.8493	1.1802	0.9223	0.8635	0.0686	0.8925	1.1417	1.0176
SbPRR95	0.9073	0.2998	1.2202	0.8925	0.9223	0.9223	0.0686	1.2408	0.9689
OsPRR1	1.1802	1.1607	0.1353	1.0866	1.0345	1.1607	1.2000	0.1080	1.2000
OsPRR37	0.9073	0.9073	1.0345	0.0121	0.3329	0.9223	0.8779	1.0515	0.2292
OsPRR73	0.8635	0.9376	1.0689	0.2757	0.1920	0.8493	0.9223	1.0515	0.4115
OsPRR59	0.1920	0.8925	1.2202	0.9223	0.8493	0.2216	0.9073	1.1802	0.9849
OsPRR95	0.9073	0.2522	1.2202	0.8925	0.9073	0.8925	0.2757	1.2202	0.9849

(c)	SbPRR73	SbPRR59	SbPRR95	OsPRR1	OsPRR37	OsPRR73	OsPRR59	OsPRR95
SbPRR73								
SbPRR59	0.8493							
SbPRR95	0.9223	0.8925						
OsPRR1	1.0345	1.1607	1.2000					
OsPRR37	0.3245	0.9223	0.8925	1.0866				
OsPRR73	0.1562	0.8493	0.9223	1.0689	0.2837			
OsPRR59	0.8353	0.1775	0.8925	1.2000	0.9223	0.8493		
OsPRR95	0.9223	0.8635	0.2522	1.1802	0.8925	0.9223	0.9073	
